# Respectful maternity care and associated factors among mothers who gave birth at public health institutions in Debre Tabor town, Northwest Ethiopia: a mixed-methods study

**DOI:** 10.3389/fgwh.2025.1513906

**Published:** 2025-01-23

**Authors:** Tadesse Ayana Fentie, Abayneh Aklilu Solomon, Mihretu Molla Enyew, Meseret Mekuriaw Beyene, Ayalew Kassie Melese, Alamirew Enyew Belay, Gebrie Getu Alemu

**Affiliations:** ^1^ Department of Midwifery, College of Health Sciences, Bahir Dar Health Science College, Bahir Dar, Ethiopia; ^2^ School of Midwifery, College of Medicine and Health Sciences, University of Gondar, Gondar, Ethiopia; ^3^ Department of Nursing, College of Health Sciences, Bahir Dar Health Science College, Bahir Dar, Ethiopia; ^4^ Department of Adult Health Nursing, College of Medicine and Health Sciences, Bahirdar University, Bahirdar, Ethiopia; ^5^ Department of Epidemiology and Biostatistics, College of Medicine and Health Sciences, University of Gondar, Gondar, Ethiopia

**Keywords:** respectful care, maternity care, health facilities, Debre Tabor town, Ethiopia

## Abstract

**Background:**

Respectful maternity care is care organized for and provided to all women in a manner that maintains their dignity, privacy, and confidentiality, ensures freedom from harm and mistreatment, and enables informed choice and continuous support during labor and childbirth. However, in many healthcare settings in Ethiopia, the standard practice of respectful obstetric care is not the norm, and a significant proportion of health professionals view patients merely as cases and do not show compassion.

**Objectives:**

To assess respectful maternity care and associated factors among mothers who gave birth at public health institutions in Debre Tabor town, Northwest Ethiopia from December 1, 2023, to January 30, 2024.

**Methods:**

An institution-based cross-sectional study was conducted using qualitative and quantitative data collection methods. Three hundred seventy participants were selected for quantitative analysis using systematic random sampling, while eight were chosen for the qualitative study through purposive sampling. The quantitative data were coded and entered into Epi Data version 4.6, then exported to the Statistical Package for the Social Sciences (SPSS) version 25 for analysis. A multivariable logistic regression analysis was performed to identify factors associated with the outcome variable. Adjusted odds ratios with 95% confidence intervals were computed to determine the significance level. Thematic analysis was used for the qualitative data.

**Results:**

The proportion of respectful maternity care was 34.1% (95%CI: 29.4, 39.2) cesarean delivery [AOR = 3.45, 95%CI: 1.60, 7.42]. Daytime delivery [AOR = 3.14, 95%CI: (1.59, 6.18)] and less than or equal to 1 day stay in a healthcare facility [AOR = 3.03, 95%CI: (1.51, 6.08)] were positively associated with respectful maternity care. Inadequate equipment and supplies, shortage of staffing, and lack of accountability were barriers to providing respectful maternity care.

**Conclusions:**

The proportion of respectful maternity care was low. Therefore, health institutions and other stakeholders should support and strengthen monitoring and evaluation mechanisms for maternal and neonatal healthcare providers and enhance education and constant support for women during their pregnancy and childbirth. Additionally, adequate access to basic equipment and supplies should be given due attention to ensure respectful maternity care.

## Background

Respectful maternity care is care organized for and provided to all women in a manner that maintains their dignity, privacy, and confidentiality, ensures freedom from harm and mistreatment, and enables informed choice and continuous support during labor and childbirth (1). However, many women worldwide continue to experience abuse and neglect during childbirth in healthcare facilities (2–4).

A study conducted in six European countries—Sweden, Norway, Belgium, Estonia, Iceland, and Denmark—found that 1 in 5 women who attended antenatal care in maternity health facilities had at least one episode of abuse (5). Several studies provide insight into the alarmingly high prevalence of serious forms of disrespect and abuse in low and middle-income countries around the world, with percentages ranging from 33.3% in Mexico (6) to 71.0% in India (7).

In contrast, a significantly higher number of women in African countries experienced disrespect and abuse (D&A) during facility-based childbirth (8). According to data from sub-Saharan African countries and Eastern African countries, approximately 44% and 46.85% of women experienced D&A during facility-based childbirth, respectively (9, 10).

According to a study in Ethiopia, the prevalence of respectful maternity care (RMC) during childbirth ranges from 12.75% to 77% (11). RMC is influenced by various factors, including the place of delivery, time of delivery, antenatal care (ANC) uptake, planning status of the index pregnancy, educational level, and obstetric complications (11–21). Promoting good interpersonal relationships and support between women and maternity staff (good reception, information provision, promptness of care, dignified care, and psychological support), and privacy and confidential care are essential to ensure respectful maternity care. Ensuring facility-based respectful maternity care (RMC) is essential for improving maternal and neonatal health, especially in sub-Saharan African countries where mortality rates and non-skilled delivery care remain high (22).

In 2019, 50% of Ethiopian women gave birth at home without the assistance of a skilled birth attendant (23). The reasons for not giving birth in a health institution included anticipated poor quality of services, a history of undesirable care from healthcare providers, a failure to adopt a patient-centered approach, and insufficient health system resources (24, 25). Recognizing these, the Ethiopian Ministry of Health has implemented RMC since 2016 as a strategic measure to increase the number of deliveries attended by skilled birth attendants (13). However, in many healthcare settings in Ethiopia, including the study area, respectful obstetric care is not yet the norm, and a significant proportion of health professionals still view patients merely as “cases” and do not show compassion (21).

Despite this, there is scant research about respectful maternity care services and associated factors during facility-based childbirth (2, 26). Moreover, the existing evidence on respectful maternity service provision has primarily been gathered from client reports (13). Providers' perspectives are needed to fully understand the barriers to providing respectful maternal care. As a result, the factors and barriers that hinder the provision of respectful maternity care during facility-based childbirth are not well known. Therefore, this mixed-methods study aims to assess respectful maternity care and associated factors among mothers who gave birth at public health institutions in Debre Tabor town, Northwest Ethiopia from December 1, 2023, to January 30, 2024

## Method and materials

### Study design and setting

A facility-based, mixed cross-sectional study, comprising a quantitative component followed by a qualitative component, was conducted from December 1, 2023, to January 30, 2024, in public health facilities in Debre Tabor town, Northwest Ethiopia. Debre Tabor is the capital of the South Gondar Zone in the Amhara regional state, located 665 km northwest of Addis Ababa, the capital of Ethiopia, and 103 km east of Lake Tana. The town has a total population of 90,081, with 42,041 (46.7%) males and 48,040 (53.3%) females. Among them, 20.96% (17,968 individuals) fall within the reproductive age range (27, 28). The town comprises nine kebeles and four public health institutions: one comprehensive specialized hospital and three health centers. These are Debre Tabor Comprehensive Specialized Hospital (DTCSH), Leul Alemayehu Health Centre (LAHC), Gaffat Health Centre (GHC), and Atse Seyife Health Centre (ASHC). These institutions provide community health care, including maternal and child health services (29).

### Source population and study population

All mothers who gave birth at public health institutions in Debre Tabor town were used as a source population. Mothers who gave birth during the data collection period were used as a study population. Moreover, senior maternal and neonatal health care (MNHC) providers were used as a study population.

### Inclusion and exclusion criteria

Mothers who gave birth at public health institutions in Debre Tabor town during the data collection period were included in the study. Mothers unable to communicate due to serious illness were excluded from the study. Moreover, senior maternal and neonatal health care (MNHC) providers were used as study participants for qualitative phase data inquiries.

### Sample size and sampling procedure

The sample size was determined using the single population proportion formula, considering a proportion of RMC at 66% (30), a 95% confidence level, and an allowed margin of error of 5%. An additional 10% was added to account for non-response, resulting in a final sample size of 380. Based on a previous delivery report, the sample was proportionally allocated to each health facility. A systematic random sampling technique was employed to recruit study participants according to their admission order. For the qualitative study, purposive sampling was used to recruit senior healthcare providers for key informant interviews (KIIs). The sample size for the qualitative component was determined based on information saturation, which was reached after eight KIIs.

## Study variables

The outcome variable for this study was the provision of respectful maternity care during childbirth. The explanatory variables included socio-demographic factors such as age, marital status, residence, religion, educational status, and occupation; obstetric factors such as antenatal care (ANC) visits, mode of delivery, parity, time of delivery, presence of neonatal and/or maternal complications, whether the pregnancy was planned or unplanned, number of ANC visits, and duration of labor; maternal health services factors such as referral status, presence of a companion, HIV status, previous use of the facility for services other than delivery, prior deliveries at the facility, and length of stay at the health facilities; and provider and facility factors, including the sex of the delivery attendant, place of delivery, place of ANC, and profession of the attendant.

### Operational definitions

Respectful maternity care was measured by 30 items, which are classified into seven categories, including the right to physical abuse-free care (8 items), the right to informed consent and information (6 items), the right to confidential care (3 items), the right to be treated with dignity (5 items), the right to be treated free of discrimination (3 items), the right not to be neglected or abandoned (3 items), and the right not to be detained or confined in health facilities (2 items). Each item has a “yes” or “no” response developed as part of the respectful maternity toolkit by the Maternal and Child Health Integrated Program (MCHIP) (3, 31). In this study, mothers were considered to be receiving respectful maternity care during childbirth if they replied “no” (which was coded as ″1″) to all questions measuring RMC, or verification criteria used for measuring the seven categories of RMC (14, 32, 33). Mothers were considered not receiving respectful maternity care if they replied “yes” (which was coded as ″0″) to one or more questions measuring RMC, or verification criteria used for measuring the seven categories of RMC (14, 32, 33).

### Data collection tools and procedures

A structured and pre-tested interview-administered questionnaire developed from previously done similar literature (14, 17, 30, 32–35) and a validated RMC checklist adopted from the Maternal and Child Health Integrated Program (MCHIP) (3, 31) was used to generate quantitative data. The tool was prepared in English and translated into the local language, Amharic, then backtranslated into English. The tool consists of five parts: the first part contains the socio-demographic factors of the respondent, the second part consists of the obstetric factors of the respondents, the third part contains maternal health service factors, the fourth part contains health facility and provider factors, and the fifth part contains categories of RMC experienced by women during facility-based childbirth. Two diploma midwives carried out the data collection process under the supervision of one BSc midwife. Postpartum exit interviews were conducted with women who had just given birth at the health facility. Additionally, some data were collected by reviewing mothers' delivery cards and interviewing healthcare providers. Each eligible woman was approached privately in a separate room from the maternity ward within the hospital grounds. After checking the completeness, the filled questionnaires were collected and signed by the supervisor. Additionally, the principal investigator provided continuous supervision and follow-up throughout the data collection period.

Following the quantitative data assessment, we collected qualitative data using a semi-structured probing guide questionnaire, which we prepared in English and translated into the local language, Amharic. One author (G.G.), a university lecturer with a master's degree who had previous experience in qualitative interview, conducts key informant interviews (KIIs). Qualitative data collection was performed using face-to-face interviews with the participants. KIIs were tape-recorded, and notes were taken. Each session of the KIIs lasted between 10 and 20 min.

## Data quality control

For quantitative data, the questionnaire was initially developed in English and translated into the local language Amharic, then back-translated into English by a different person to check the consistency. Supervisors and data collectors underwent 1-day training on the study objectives, obtaining informed consent, and approaching the participant. Continuous data monitoring was conducted by supervisors and the principal investigator throughout the study. To ensure the trustworthiness of the qualitative data, the criteria from Lincoln and Guba's framework were applied. For dependability, detailed reporting of the research process was carried out; for conformability, an independent person checked interview transcriptions; and transferability was established using the purposive sampling technique.

## Data processing and analysis

For quantitative data, after checking completeness, the data were coded and entered into Epi Data version 4.6 and then exported to SPSS version 25 for cleaning and analysis. Descriptive summary measures, such as frequency, percentages, mean, and standard deviation, were employed to describe the characteristics of the respondents. Variance Inflation Factor (VIF) was used to check for multi-colinearity among predictors. The model fitness was checked using the Hosmer and Lemeshow goodness of fitness test, yielding a value of 0.842. Binary logistic regression analyses were carried out to assess the association between each independent variable and the outcome variable. Variables with a *p*-value < 0.25 were entered into a multivariable logistic regression model, and the selected variables were entered sequentially by using backward stepwise regression to control for potential confounders and identify predictors of the outcome variable. An association between outcome and explanatory variables was presented using an adjusted OR with a 95% confidence interval. Variables with a *p*-value < 0.05 were considered statistically significant.

Qualitative data were transcribed, translated, coded, and analyzed using Qualitative Data Analysis (QDA) minor lite version 2.0.3. A thematic approach was utilized.

## Results

### Quantitative part

## Socio-demographic characteristics of the participants

This study involved 370 participants, resulting in a response rate of 97.4%. The mean ± SD of respondents' age was 29.04 ± 3.98 years. The minimum and maximum ages of the respondents were 19 and 37 years. The majority (68.6%) fall into the age category of 25–34 years. Approximately two-thirds (67.6%) of the participants were Orthodox. Half the participants had no formal education, and about 58.9% were from rural areas. Most (93.5%) of the study participants were married, and the majority (60.3%) were housewives (Table 1).

**Table 1:** Socio-demographic characteristics of mothers who gave birth at public health institutions in Debre Tabor town, Northwest Ethiopia, 2024 (*n* = 370).

Variables	Category	Frequency	Percentage
Age	<25	74	20.0
	25–34	254	68.6
	≥35	42	11.4
Place of residence	Urban	152	41.1
	Rural	218	58.9
Marital status	Married	346	93.5
	Divorced	11	3.0
	Single	13	3.5
Educational status	Have no formal education	187	50.5
	Primary education (1–8)	59	15.9
	Secondary education (9–12)	29	7.9
	College and above	95	25.7
Religion	Orthodox	250	67.6
	Muslim	97	26.2
	Protestant	23	6.2
Occupation	Housewife	223	60.3
	Government employee	69	18.6
	Merchant	56	15.1
	Private employee	11	3.0
	Student	11	3.0

## Obstetric factors

Out of 370 participants, the majority (84.3%) were multiparous. While 95.4% had at least one antenatal care (ANC) visit during their index pregnancy, only 22.2% attended four or more ANC visits. Notably, a significant portion (81.6%) of pregnancies among participants were intended. Nearly half (44.9%) gave birth during nighttime hours. The majority (60.3%) did not encounter complications during labor and delivery. Furthermore, a substantial proportion (89.2%) experienced a labor duration of less than 12 h. Of the total respondents, 74.1% gave birth via spontaneous vaginal delivery (Table 2).

**Table 2:** Obstetric characteristics of mothers who gave birth at public health institutions in Debre Tabor town, Northwest Ethiopia, 2024 (*n* = 370).

Variables	Category	Frequency	Percentage
Parity	Primiparous	58	15.7
	Multiparous	312	84.3
ANC Visit	Yes	353	95.4
	No	17	4.6
Number of ANC visit	<4	288	77.8
	≥4	82	22.2
Pregnancy planned or unplanned	Planned	302	81.6
	Unplanned	68	18.4
Delivery time	Day	204	55.1
	Night	166	44.9
Mode of delivery	SVD	274	74.1
	Vacuum or forceps	36	9.7
	Cesarean	60	16.2
Duration of labor	<12 h	330	89.2
	≥12 h	40	10.8
Any maternal/fetal complication	Yes	147	39.7
	No	223	60.3

## Maternal health service-related factors

Out of the 370 participants, 51.1% arrived directly for labor and delivery, while 52.2% had previous childbirth experiences within health facilities. Additionally, 55.4% of participants stayed in the health facility for 24 h or more. The majority (69.7%) utilized health facility services beyond mere delivery. Notably, 96.2% of participants were negative for HIV. Furthermore, half (50.3%) of the participants had companions during the labor process (Table 3).

**Table 3:** Health service history of mothers who gave birth at public health institutions in Debre Tabor town, Northwest Ethiopia, 2024 (*n* = 370).

Variables	Category	Frequency	Percentage
Referral status	Referred	189	51.1
	Came directly	181	48.9
HIV status	Positive	14	3.8
	Negative	356	96.2
Previously using the Facility other than delivery	Yes	258	69.7
	No	112	30.3
Previously history of delivery at the health facility	Yes	193	52.2
	No	177	47.8
Duration of stay at health facility	≤1 day	165	44.6
	>1 day	205	55.4
Have companion	Yes	186	50.3
	No	184	49.7

## Health facility and provider factors

Out of the 370 respondents, 89.2% gave birth in a hospital setting. Midwives managed 53.3% of the deliveries, while 43.8% of the participants were attended by female healthcare providers. Nearly two-thirds (64.1%) of participants received antenatal care at health centers (Table 4).

**Table 4:** Health facility and provider factors of mothers who gave birth at public health institutions in Debre Tabor town, Northwest Ethiopia, 2024 (*n* = 370).

Variables	Category	Frequency	Percentage
Sex of delivery attendant	Male	208	56.2
	Female	162	43.8
Profession of delivery attendant	Midwife	196	53.0
	Doctor	93	25.1
	IESO	34	9.2
	Medical Intern	47	12.7
Place of antenatal care	Health center	237	64.1
	Hospital	133	35.9
Place of delivery	Health center	40	10.8
	Hospital	330	89.2

## Proportion of respectful maternity care

The overall proportion of respectful maternity care provision among laboring mothers in the health facilities of Debre Tabor town was 34.1% (95% CI: 29.2, 39.1). The most common category of respectful maternity care identified by women in this study was providing detention-free care, which was fully respected, followed by almost all (99% and 94%) of the participants receiving discrimination-free care and abandonment-free care, respectively (Table 5).

**Table 5:** Respectful maternity care categories among mothers who gave birth at public health institutions in Debre Tabor town, Northwest Ethiopia, 2024 (*n* = 370).

Category	Verification criteria	Yes%	No%
The right to Physical abuse-free care	The health care providers used physical forces (slapping, pinching, beating/hitting) me while I was in labor pain	1 (0.3)	369 (99.7)
	The birth attendant(s) threatened me with beating to let me obey their order	1 (0.3)	369 (99.7)
	The healthcare provider(s) suture my perineum without the use of local anesthesia	5 (1.4)	305 (82.4)
	My leg was tied down on a delivery bed when I was in the delivery	0 (0.0)	370 (100)
	The healthcare provider did not allow me to assume my position of choice during labor and delivery	108 (29.2)	262 (70.8)
	The healthcare provide did not allow me to ambulate during the labor without reason	112 (30.3)	258 (69.7)
	The healthcare provider pushed my tummy down to deliver the baby (used fundal pressure)	8 (2.2)	302 (81.6)
	I was denied food or fluid while I was in labor without medically necessitated	10 (2.7)	360 (97.3)
	Total	34%	66%
Right to informed consent	The healthcare provider did not introduce himself/herself to me and my companion	222 (60)	148 (40)
	The healthcare providers did not share the findings of my initial assessment for me and or my families	88 (23.8)	282 (76.2)
	The healthcare provider did not encourage me to ask questions	86 (23.2)	284 (76.8)
	The healthcare providers did not explain to me what was being done and what to expect throughout the labor and birth process	85 (23.0)	285 (77.0)
	The healthcare provider did not obtain my consent or permission before any procedure	30 (8.1)	340 (91.9)
	The healthcare providers coerce me to undergo C/S	1 (0.3)	369 (99.7)
	Total	61.4%	38.6%
Right to confidentiality	The healthcare provider did not use drapes or other visual barriers to protect my privacy	82 (22.2)	288 (77.8)
	The healthcare providers allowed another person to the room while I was giving birth who could observe me while I was naked on the bed	6 (1.6)	364 (98.4)
	The healthcare providers discuss my private health information in a way that others can hear	0 (0.0)	370 (100)
	Total	23%	77%
Right to be treated with dignity	The healthcare provider did not speak to me politely throughout the labor	14 (3.8)	356 (96.2)
	The healthcare provider intimidated/humiliated me at least one time	0 (0.0)	370 (100)
	The healthcare providers make negative comments during labor	0 (0.0)	370 (100)
	The health care providers shout at or scold me during labor pain	0 (0.0)	370 (100)
	The healthcare providers did not allow my companion to enter the delivery room	90 (24.3)	280 (75.7)
	Total	26%	74%
Right to be free of discrimination	The healthcare providers discriminate by race, ethnicity, economic status, or poor educational status, rural areas come	0 (0.0)	370 (100)
	The healthcare providers discriminate because of being a teenager or advanced age	0 (0.0)	370 (100)
	The healthcare providers discriminate because of being HIV-positive	4 (1.1)	11 (3.0)
	Total	1%	99%
Right not to be abandoned or neglected	The healthcare provider left me alone or unattended	4 (1.1)	366 (98.9)
	I gave birth in the health institution by myself because the care providers were not around me	2 (0.5)	368 (99.5)
	The healthcare provider did not come quickly when I called him/her	18 (4.9)	352 (95.1)
	Total	6%	94%
Right not to be detained or confined	Discharge was postponed until hospital bills were paid	0 (0.0)	370 (100)
	I was detained in a health facility against my will	0 (0.0)	370 (100)
	Total	00	100%

## Factors associated with respectful maternity care

In the Bivariable logistic regression, 13 variables with a *p*-value less than 0.25 were considered for multivariable analysis. These variables included place of residence, educational status, occupation of mothers, mode of delivery, pregnancy wanted/intended, referral from another health facility, number of ANC visits, delivery time, sex of health provider, having a birth companion, previous delivery at a health facility, previously using the facility other than delivery, and length of stay in a health facility. The results of the multivariable analysis indicated that cesarean delivery, time of delivery, and length of stay in a health facility were found to have statistical associations with respectful maternity care.

This study identified that those mothers who gave birth by cesarean section were 3.45 times [AOR = 3.45, 95% CI: 1.60, 7.42] more likely to get respectful maternity care compared to those who gave birth by spontaneous vaginal delivery.

This study indicated that those mothers who gave birth at daytime were 3.14 times [AOR = 3.14, 95% CI: 1.59, 6.18) more likely to receive respectful maternity care as compared to those who gave birth at nighttime.

The odds of receiving respectful maternity care were 3.03 times [AOR = 3.03, 95% CI: 1.51, 6.08)] higher among mothers who stayed less than or equal to 1 day compared to those who stayed longer in health facilities (Table 6).

**Table 6:** Factors associated with respectful maternity care among mothers who gave birth at public health institutions in Debre Tabor town, Northwest Ethiopia, 2024 (*n* = 370).

Variables	RMC		COR (95% CI) *n* = 370	AOR (95% CI) *n* = 370	***P***
	Yes	No			
Place of residence					
Urban	75	77	3.18 (2.04, 4.98)	0.73 (0.37, 1.41)	0.353
Rural	51	167	1	1	
Educational status					
No formal education	46	163	1	1	
Primary and secondary education	41	45	3.22 (1.89, 5.51)	1.475 (0.73, 2.95)	0.272
College and above	39	36	3.83 (2.19, 6.71)	1.357 (0.55, 3.30)	0.502
Occupation					
Housewife	57	166	1	1	
Government employee	33	36	2.67 (1.52, 4.67)	1.20 (0.62, 2.33)	0.577
Others^a^	36	42	2.49 (1.45, 4.27)	1.67 (0.89, 3.16)	0.109
Pregnancy wanted/intended					
Planned	114	188	2.83 (1.45, 5.50)	0.679 (0.28, 1.60)	0.379
Un planned	12	56	1	1	
Mode of delivery					
Vaginally	95	215	1	1	
Cesarean	31	29	2.41 (1.38, 4.23)	3.45 (1.60, 7.42)*	0.002
Time of delivery					
Day	107	97	8.53 (4.91, 14.81)	3.14 (1.59, 6.18)*	0.001
Night	19	147	1	1	
Number of ANC visit					
<4	76	212	1	1	
≥4	50	32	4.35 (2.60, 7.29)	1.64 (0.92, 2.94)	0.093
Having birth companion					
Yes	98	88	6.20 (3.78, 10.17)	1.84 (0.98, 3.43)	0.056
No	28	156	1	1	
Length of stay in health facility					
≤1 day	86	79	4.49 (2.83, 7.12)	3.03 (1.51, 6.08)*	0.002
>1day	40	165	1	1	
Previous delivery at health facility					
Yes	100	93	6.24 (3.77, 10.32)	1.26 (0.59, 2.69)	0.535
No	26	151	1	1	
Previously using the health facility other than delivery					
Yes	111	147	4.88 (2.68, 8.87)	0.94 (0.38, 2.42)	0.946
No	15	97	1	1	
Referred from other health facility					
Yes	57	132	0.70 (0.45, 1.07)	0.76 (0.44, 1.29)	0.310
No	69	112	1	1	
Sex of the health provider					
Male	102	106	5.53 (3.31, 9.22)	1.61 (0.84, 3.08)	0.152
Female	24	138	1	1	

AOR, adjusted odds ratio; COR, crude odds ratio.

^a^ Others student and private employee; RMC, respectful maternity care; 1, Reference.

* (*P*-value < 0.05) in multivariable analysis.

## Qualitative part

### Barriers to the provision of respectful maternity care

Barriers to respectful maternity care during childbirth from health care providers' perspectives resulted in three main themes: facility-related barriers, health care provider-related barriers, maternal-related barriers, and nine sub-themes. Inadequate health facility equipment and supply, lack of accountability, lack of professional advancement, staffing shortages, task-oriented approach, insufficient nighttime staffing, lack of maternal health education, maternal perceived neglect during early labor, and presence of maternal and fetal complications (Table 7).

**Table 7:** The main themes and sub-themes emerged from key informants at public health institutions in Debre Tabor town, Northwest Ethiopia, 2024 (*n* = 8).

The number of instances each sub-theme came up during the Interviews (*n* = 8)	Sub-themes	Main Themes
10	Inadequate health facility equipment and supply	Facility-related barriers
11	Lack of accountability	
10	Lack of professional advancement	Provider-related barriers
7	Staffing shortages	
2	Task-oriented Approach	
3	Insufficient Nighttime Staffing	
6	Lack of maternal health education	Maternal-related barriers
1	Maternal Perceived Neglect during Early Labor	
6	Presence of maternal and fetal complications	

## Main theme: facility-related barriers

This theme encompasses barriers within healthcare facilities that impede respectful maternity care. It includes deficiencies in facility resources and a lack of accountability practices that impact the quality of care.

## Sub-theme: inadequate health facility equipment and supply

Inadequate or absence of essential medical equipment, and supplies, can severely impact the quality of care provided, increasing the risk of occupational hazards for healthcare providers and potentially leading to suboptimal or disrespectful treatment of patients.

“Health facilities were not always fully equipped with all necessary equipment, such as screens, and there was often a shortage of personal protective equipment, leading to a risk of exposure to blood splashes, which might, in turn, result in disrespectful care.” (KII7)

## Sub-theme: lack of accountability

The absence or insufficiency of mechanisms and practices that ensure healthcare providers are held responsible for their actions and behaviors. This leads to disrespectful behaviors towards patients and escalating negative behaviors, as individuals may feel emboldened to act without fear of consequences.

“I believe that the lack of accountability is a pivotal factor contributing to disrespectful or hurtful behavior towards others. When individuals are not held accountable, the potential for the escalation of misconduct is significantly heightened.”(KII6)

## Main themes: healthcare provider-related barriers

This theme encompasses barriers related to healthcare providers that impede the provision of respectful maternity care. These barriers stem from issues within the professional environment of healthcare providers, affecting their performance, satisfaction, and ability to deliver high-quality, respectful care to patients.

## Sub-themes: lack of professional advancement

Insufficient opportunities for career growth, promotions, and continuing education for healthcare providers. When healthcare professionals are not recognized for their experience and dedication through promotions or opportunities to further their education, it can lead to dissatisfaction and demoralization. Over time, this can erode their passion for the profession and negatively affect their performance and commitment to patient care.

“Despite eight years of service, during which I have earned only 8,000 Birr, I have not received any promotions or educational opportunities. This lack of professional advancement has led to dissatisfaction, causing a gradual decline in my passion for the profession, consequently affecting the quality of service provided to mothers and resulting in a diminished level of dedication and commitment. It is imperative to address these underlying issues to ensure the provision of complete and comprehensive care to mothers.” (KII1)

“Certain service providers might lack adequate training, potentially resulting in the delivery of services that fall below the expected or desired quality.” (KII5)

## Sub-themes: staffing shortages

When there are not enough midwife professionals to adequately cover all necessary responsibilities, the quality and safety of patient care can be compromised. This shortage of skilled staff can lead to increased workloads, burnout among existing professionals, and a decline in the standard of care provided.

“In this hospital, there are seven beds in the intrapartum ward, but only three midwife professionals are assigned. The remaining responsibilities are covered by students and university staff who may not possess the requisite expertise. This situation poses a significant challenge in ensuring the provision of respectful maternal health services.” (KII8)

## Sub-themes: task-oriented approach

A focus on completing specific responsibilities and tasks, often with the primary goal of preventing complications, sometimes at the expense of considering broader ethical or moral implications. While this approach can be effective in preventing adverse outcomes, it may limit the scope for compassionate and individualized patient care, potentially leading to a more mechanistic and less empathetic healthcare environment.

“My emphasis lies in fulfilling my responsibilities; instead of dwelling on notions of right or wrong, our primary focus is to preemptively prevent any complications from arising.” (KII3)

## Sub-theme: insufficient nighttime staffing

Inadequate number of healthcare professionals available during night shifts, where a limited number of staff must manage a high volume of patients, leading to significant strain and fatigue. This exhaustion can impede their respectful and thorough attention to each patient.

“I experience fatigue, particularly during nighttime, as the presence of only three midwives becomes challenging when faced with an average of ten to twelve mothers in need of delivery. This overwhelming situation hinders my ability to provide respectful care.” (KII2)

### Main theme: maternal-related barriers

This theme encompasses aspects of maternal health, knowledge, and perception that impede the provision of respectful maternity care. These barriers stem from gaps in maternal education, perceived neglect during labor, and complications affecting maternal and fetal health, all of which impact the quality and experience of maternity care.

## Sub-themes: lack of maternal health education

The gap in knowledge and understanding among expectant mothers regarding the natural processes of labor and childbirth often leads to a more negative experience and perception of the care received.

“In our community, many of our mothers are uneducated; they consider that they give birth as soon as they arrive at the hospital because they do not understand the natural process of labor.” (KII4)

## Sub-themes: maternal perceived neglect during early labor

The feelings of abandonment and inadequate attention experienced by mothers in the initial stage of labor due to infrequent monitoring and interaction from healthcare providers lead to feelings of neglect and abandonment among early laboring mothers, leading to a negative perception of the care received.

“Mothers who are in the first stage of labor sometimes feel like they have not been treated well. After providing them with a bed and conducting an initial examination, we typically check on them every hour unless they have an obstetric issue. However, we tend to check on mothers who are co-sleeping every thirty minutes or every fifteen minutes, which can make the first stage of laboring mothers feel neglected. As a result, mothers in the first stage of labor may feel like they have been abandoned.” (KII4)

## Sub-themes: presence of maternal and fetal complications

In the presence of maternal or fetal complications, healthcare providers are committed to preserving lives through any necessary means. Any complication affecting either the mother or newborn can present a substantial barrier to providing respectful delivery services.

“In certain situations, a patient's life or well-being might be at risk, necessitating decisive action even if it contradicts the patient's wishes. For instance, in the event of severe bleeding, immediate intervention may be required to save their life, even if the patient opposes such measures. Striking a delicate balance between respecting the client's autonomy and taking essential steps to safeguard their health is imperative in such critical scenarios.” (KII5)

## Discussion

The proportion of respectful maternity care was 34.1%. This finding is consistent with studies conducted in Harar, 38.4%, and West Shewa, Ethiopia, 35.8% (15, 32). However, this finding is higher than studies conducted in Benishangul Gumuz Region (12.6%), Addis Ababa (24.6%), and Arba Minch town, Ethiopia (1.1%) (13, 36, 37). This might be due to variations in the study period, healthcare practices, and cultural norms within different regions or settings. Additionally, this disparity might be due to the exclusion of mothers who underwent emergency cesarean sections in a study carried out in Addis Ababa. However, this result is lower than that of research conducted in Tanzania (85%), India (71%), and Kenya (81%) (38–40). This might be variations in research periods, socioeconomic characteristics, and access to maternal and child health services because the quality of care is significantly affected when an institution's infrastructure, personnel, supplies, and equipment do not meet recognized standards of care (41) This study indicated that mothers who gave birth during the day were 3.14 times more likely to receive respectful maternity care than mothers who gave birth at night. This finding is consistent with studies conducted in Kenya, West Shewa, and Debre Birhan, Ethiopia (32, 40, 42). One reason for this discrepancy may be that healthcare providers offer more respectful care during the day when they are more likely to be supervised by senior staff and managers. Additionally, more staff members and better resources are often available during daytime hours. In contrast, mothers giving birth at night may experience less respectful care due to a smaller night-shift staff and the resulting heavier workload. Fatigue from disrupted sleep schedules may also contribute to these differences in care quality between day and night shifts.

This study identified that those mothers who gave birth by cesarean section were 3.45 times more likely to get respectful maternity care compared to those who gave birth by spontaneous vaginal delivery. This finding is consistent with studies conducted in the Hadiya zone, Ethiopia, and Rwanda (43, 44). This might be because mothers who gave birth through cesarean section might have received special attention and support from healthcare providers due to concerns regarding potential complications associated with the surgical procedure. This increased attention could lead to a more positive experience and perception of the care received. This finding contrasts with studies conducted in Bahirdar and Arba Minch, Ethiopia (17, 36). This might be explained by a study in Arbaminch town, Ethiopia, which excluded mothers who underwent elective cesarean sections. Furthermore, this discrepancy may be explained by differences in cultural norms and healthcare practices within different regions or settings.

The odds of receiving respectful maternity care were 3.03 times higher among mothers who stayed less than or equal to one day compared to those who stayed longer in health facilities. This finding is consistent with studies conducted in Tanzania, Bahirdar town, Ethiopia, and Nepal (33, 34, 45). This might be because mothers who stayed for a shorter period may have better continuity of care. With consistent support from healthcare providers throughout their stay, this continuity of care can contribute to a more positive experience and perception of respectful maternity care. This finding contrasts with a study conducted in West Shewa, Ethiopia (32). This might be due to differences in a study setting, staffing, and resources.

## Strengths and limitations of the study

The strength of this study is the triangulation of quantitative with qualitative inquiry to get robust data in determining factors affecting RMC services Despite these strengths, the limitation of this study was the use of nurses as data collectors and interviews in hospital settings, which might introduce bias due to its sensitivity nature of the study.

## Conclusions

As per this study, the proportion of respectful maternity care in public health facilities in Debre Tabor town was low. Factors such as giving birth in the daytime, cesarean delivery, and less than or equal to 1 day stay in a healthcare facility were positively associated with RMC. Additionally; Inadequate equipment and supplies, lack of accountability mechanisms, lack of professional advancement, Health care provider shortages, Task-oriented Approach, lack of maternal Health education, Maternal Perceived Neglect during early labor, and the presence of maternal and fetal complications were barriers to the provision of respectful maternity care service. Therefore, health institutions and other stakeholders should support and reinforce monitoring and evaluation mechanisms for MNHC providers and enhance education and constant support for women during their pregnancy and childbirth. Additionally, access to basic equipment and supplies should be given due attention to ensure respectful maternity care.

## Data Availability

The datasets presented in this study can be found in online repositories. The names of the repository/repositories and accession number(s) can be found in the article/Supplementary Material.
