# Supplementary material for: Respectful maternity care and associated factors among mothers who gave birth at public health institutions in Debre Tabor town, Northwest Ethiopia: a mixed-methods study
Source: Front Glob Womens Health. 2025 Jan 23;6:1513906. doi: 10.3389/fgwh.2025.1513906 (PMC11798984; doi:10.3389/fgwh.2025.1513906)
Supplement: Supplementary file 1 [file Datasheet1.pdf]

Annex I: English version Information Sheet

My name is ___________________________ I am working as a data collector in the research conducted by Tadesse Ayana who is conducting this research for the partial fulfillment of his Master's degree at Gondar University College of Medicine and Health Science School of Midwifery Department of Clinical Midwifery. We are trying to assess the respectful maternity care of women during facility childbirth. We would like your honest opinion about the questions especially what you had experienced disrespect and abuse by health professionals during giving birth in a health facility.

Name of the organization: Gondar University College of Medicine and Health Science School of Midwifery Department of Clinical Midwifery.

Name of the Sponsor: Gondar University

Introduction: An information sheet and consent form are prepared for mothers who give birth in a health facility, both quantitative and qualitative cross-sectional studies will be used to assess respectful maternity care of women during childbirth at the public health facility of Debre Tabor town.

Purpose: I am hopeful that this research will benefit all pregnant and laboring mothers including newborn health care improvement and quality of care. I will provide research results to the concerned body for intervention.

Procedure: To assess the respectful maternity care of women during childbirth in Debre Tabor town you are invited to take part in this project. If you are willing to participate in this project, you need to understand and say „yes‟ on the agreement form. Then after, you will be interviewed by the data collector. All your responses and the results obtained will be kept confidential by using a coding system whereby no one will have access to your response.

Risk/ Discomfort: By participating in this research project, you may feel that it has some discomfort, especially spending time about 30 minutes. We hope you will participate in the study for the sake of the Benefit of the research result. I am sure there is no risk in participating in this research project.

Benefits There may not be direct benefits to you but your Participation is likely to help us in the assessment of the status of respectful maternity care during childhood birth ultimately,

This will help us to identify the gap and take the appropriate intervention by the authorized stakeholder. You will not be provided any incentive or payment to take part in this project.

Confidentiality: The information collected from this research project will be kept confidential and information about you that will be collected by this study will be stored in a file, without your name, but a code number assigned to it. In addition, it will not be revealed to anyone except the principal investigator and will be kept locked with a key.

Right to refuse or withdraw: You have full right to refuse to participate in this research. You can choose not to respond to some or all questions if you do not want to give your response. You have also the full right to withdraw from this study at any time you wish, without losing any of your rights. If you have any questions, you can ask at any time. If you have additional questions about the study please contact

May I continue the interview? Yes ☐ No ☐

Name of advisors

Mr. Abayneh Aklilu (MSc, asst. Professor)

Tel: +251-918-77-14-33

E-mail: sabayneh7@gmail.com

Mr. Mihretu Molla (MSC, asst. Professor)

Tel: +251-918-29-85-19

E-mail: mihretumolla143@gmail.com

Name of investigator

Tadesse Ayana

Tel: +251-920-77-48-211

Email: tadesseay6@gmail.com

Annex II: English version Consent form

I was selected as a participant who heard the information in the consent sheet and understood what is required of me and what will happen to me if I take part in the study. I understand that all the information regarding me, like my name and all answers given by me, will not be transferred to the third party. I also understood that I could withdraw from the study at any time without giving a reason and without my or my family's being affected by my refusal.

The Participant:

1. Agreed ☐

2. Did not agree to end the interview and thank the responde ☐

Interviewer Agreement

I certify that I have taken written consent from the respondent that she agreed to participate in The study and I have confirmed the agreement is correct.

Interviewer Name: ____________________________ Signature_____________________________

Date |________________ month |_____________| 2024.

Supervisor Name: ____ Signature__________________

Date |________________month |_____________| 2024.

Type of facility: ----------------------------------

Mother's code……

|________|_______|________| Date |________|________| 2024

Interviewer's code |________|________|

Start Time __________ End time_________

Annex III: English version questionnaire

Identification code ___________

**Part I: Sociodemographic characteristics of the mothers**				
**S.no**	**Question**	**Response**	**Skip**	**Code**
101	How old are you in a completed year?	1. ------year		
102	What is your Marital status?	1. Single<br>2. Currently married<br>3. divorced<br>4. widowed		
103	Where is your residential area?	1. Urban<br>2. Rural		
104	What is your religion?	1. Orthodox<br>2. Muslim<br>3. Protestant<br>4. Catholic<br>5. Other (specify)-----------		
105	What is the highest level of education you have completed?	1. Unable to read and write<br>2. Only read and write<br>3. Primary education (1-8)<br>4. Secondary education (9-12)<br>5. College and above		
106	What is your current occupation?	1. Government employee<br>2. Merchant<br>3. Private employee<br>4. Student<br>5. Housewife<br>6. Other (specify)----------		
**Part II: Obstetric characteristics of the mothers**				
**S.no**	**Question**	**Response**	**Skip**	**Code**
201	Did you have an antenatal care visit for the current pregnancy?	1. Yes<br>2. No	If No skip to Q 203	

202	If yes to Q 207, how many visits did you have?	1……. in number		
203	Was the pregnancy wanted?	1. Yes<br>2. No		
204	How many total births have you given so far (including stillbirth and current pregnancy?	1. …… in number		
205	What was your mode of delivery	1. Spontaneous vaginal birth<br>2. Vacuum /forceps<br>3. Cesarean		
206	Have you faced any complications during labor?	1. Yes<br>2. No	If No skip to Q 208	
207	If yes to Q 206, what was the complication that you face	1. Hemorrhage<br>2. Hypertensive disorder<br>3. Perineal tear<br>4. Other (specify)------------		
208	Has your neonate faced any complications?	1. Yes<br>2.No	If No skip to Q 210	
209	If yes to Q 208, what was the complication that your neonate faced?	1. Distress<br>2. Mal presentation<br>3. Physical injury<br>4. Others (Specify)-----------		
210	What was the time you gave birth?	1. Day<br>2. Night		
**Maternal Health service-related factors**				
**S.no**	**Question**	**Response**	**Skip**	**Code**
301	Did you have a companion/supporter while you were in labor?	1. Yes<br>2. No		
302	Did you have previously delivered at this facility?	1. Yes<br>2. No		
303	Did you previously use the facility other than delivery?	1. Yes<br>2. No		
304	Are you referred from another health facility?	1. Yes<br>2. No		

305	For how many days did you stay in this facility?	1.---------in days		
306	Have you been screened for HIV during antenatal care or delivery?	1. Yes<br>2. No	If No skip to Q 401	
307	If yes what was your test result? (See her delivery cared)?	1. Reactive<br>2. Non-reactive<br>3. Unknown		
**Health facility and provider-related factors**				
**S.no**	**Question**	**Response**	**Skip**	**Code**
401	What type of health facility did you receive ANC follow-up?	1. Health center<br>2. Hospital<br>3. Private clinic		
402	What type of health facility did you give birth to?	1. Health center<br>2. Hospital		
403	What was the sex of the professionals that assisted you? (see the document)	1. Male<br>2. Female		
404	The profession of her birth attendant? (see the document)	1. IESO<br>2. Midwifery<br>3. Health officer<br>4. Doctor<br>5. Medical intern<br>6. Others (specify)------------		
**Part III: Categories and types of respectful maternity Care women experience during childbirth in a health facility**				
**S.no**	**Question**	**Response**	**Skip**	**Code**
The right to Physical abuse-free care				
301	Did the health care providers use physical force (slapping, pinching, beating /hitting) against you while you were in labor pain?	1. Yes<br>2. No		
302	Did the birth attendant(s) threaten you with beating to let you obey their order	1. Yes<br>2. No		
303	Did the health care provider(s) suture your perineum without the use of local anesthesia?	1. Yes<br>2. No<br>3. NA		
304	Did your leg was tied down on a delivery bed when you were in delivery?	1. Yes<br>2. No		

305	Did the healthcare provider not allow you to assume your position of choice during labor and delivery?	1. Yes<br>2. No		
306	Did the healthcare provider not allow you to ambulate during the labor without reason?	1. Yes<br>2. No		
307	Did the birth attendant push your tummy down to deliver the baby (used fundal pressure)?	1. Yes<br>2. No<br>3. NA		
308	Have you been denied food or fluids in labor unless medically necessitated?	1. Yes<br>2. No		
Right to informed consent				
309	Did the healthcare provider not introduce himself/herself to you and your companion?	1. Yes<br>2. No		
310	Did the healthcare providers not share the findings of your initial assessment with you and or your families?	1. Yes<br>2. No		
311	Did the healthcare provider not encourage you to ask questions	1. Yes<br>2. No		
312	Did the healthcare providers not explain to you what is being done and what to expect throughout the labor and birth process?	1. Yes<br>2. No		
313	Did the healthcare provider not obtain your consent or permission before any procedure?	1. Yes<br>2. No		
314	Did the health care providers coerce you to undergo C/S?	1. Yes<br>2. No		
Right to confidentiality				
315	Did the healthcare provider not use drapes or other visual barriers to protect privacy?	1. Yes<br>2. No		
316	Did the healthcare providers allow another person to the room where you were giving birth who could observe you while you were naked on the bed?	1. Yes<br>2. No		
317	Did the healthcare providers discuss your private health information in a way that others could hear?	1. Yes<br>2. No		
Right to be treated with dignity				
318	Did the healthcare provider not speak to you politely throughout the labor?	1. Yes<br>2. No		
319	Did the healthcare provider intimidate/ humiliate you at least one time?	1. Yes<br>2. No		
320	Did Healthcare providers make negative comments during the labor?	1. Yes<br>2. No		

321	Did the healthcare providers shout at or scold you during labor pain?	1. Yes<br>2. No		
322	Did the healthcare providers not allow your companion to enter the delivery room?	1. Yes<br>2. No		
Right to be free of discrimination				
323	Did the healthcare providers discriminate against you by race, ethnicity, economic status, or poor educational status, in rural areas come?	1. Yes<br>2. No		
324	Did the health care providers discriminate against you because of being a teenager or an advanced age?	1. Yes<br>2. No		
325	Did the health care providers discriminate against you because of being HIV-positive?	1. Yes<br>2. No<br>3. NA		
Right not to be abandoned or neglected				
326	Did the health care provider leave you alone or unattended?	1. Yes<br>2. No		
327	Have you given birth in the health institution by yourself because the care providers were not around you?	1. Yes<br>2. No		
328	Did the healthcare provider not come quickly when you called him/her?	es<br>o		
Right not to be detained or confined				
329	Was your discharge has been postponed until hospital bills were paid?	1. Yes<br>2. No		
330	Have you been detained in a health facility against your will?	1. Yes<br>2. No		

Thank You for Your Time and Participation!!!

Annex IV: English version Information and consent form for key informant interview

Dear respondent! Good morning/good afternoon my name is …………………………..and I am collecting data on respectful maternity care provision and associated factors among mothers who gave birth at DebreTabor town public health facilities. You are selected purposively and your names will not be written in this format. Whatever information you provide will be kept strictly confidential, and will not be shared with anyone else. I am going to ask you some questions that are not difficult to answer and last about 30 minutes The conversation will be taped only with your permission; however, after we transcribe the audio data into textual form the taped data will be erased and you have a right to participate or not, or discontinue at any time. However, your honest answers to these questions will be very helpful for the success of the research.

Are you willing to participate in the interview?

Agree ☐ Decline ☐

Interviewer: Name ______________________ Signature __________

Annex V: English version Semi-structured interview guide

Socio-demographic characteristics of the KIIs

S.no	Character	
1	Age in years	
2	level of education	
3	Work experience in year	
4	Position	

1. Do you share your thoughts on what respectful maternity care means to you? How would you rate the quality of respectful maternity care service provided in this health facility? Can you explain the idea behind your answer?
2. Have you ever found yourself in a situation where you felt like you acted in a way that respected women during childbirth? If so, can you elaborate on when and how it happened?
3. What do you believe are the key factors that impact the provision of respectful delivery service? Can you explain in detail?
4. From your perspective, do you think that the issue of respectful maternal care has been adequately addressed? Can you elaborate on how you came to this conclusion?
5. Lastly, do you have any additional ideas or thoughts on the topic that you believe is crucial and should be discussed?

Thank you for your time and participation!!

አባሪVI: የአማርኛ ቅጅ የጥናቱ መረጃ ቅፅ

ጤና ይስጥልኝ ፡ ስሜ -------------------------------ይባላል ፡ በጎንደር ዩኒቨርሲቲ ፣ህክምናና ጤና ሳይንስ ኮሌጅ በሚድዋይፈሪ ትምህርት ክፍል የድህረ ምረቃ (የሁለተኛ ድግሪ) ተማሪ የሆኑት አቶ ታደሰ አያና የዚህ ጥናት ዋና ተመራማሪ ሲሆኑ እኔ ደግሞ የጥናቱ መረጃ ሰብሳቢነኝ፡፡ የዚህ ጥናት አላማም በመንግስት ጤና ተቋማት የሚወልዱ እናቶች በምጥ እና በወሊድ ጊዜ የሚያጋጥማቸዉን አክብሮት የጎደለዉ እና እንግልት የበዛበት የወሊድ አገልግሎት ምን እንደሚመስል እና ተያያዥ ምክንያቶች ላይ ጥናት በማድረግ የማሻሻያ መንገዶችን መጠቆም ነዉ ፡፡ እርስዎ በዚህ ጥናት ላይ እንዲሳተፉ የተመረጡ ሲሆን የሚሰጡን ማንኛዉም መረጃ ሚስጢራዊነቱ የተጠበቀ ይሆናል፡፡ ተሳትፎዎ ሙሉበሙሉ በእርሶዎ ፈቃደኝነት ላይ ብቻ የተመሰረተ ይሆናል ፡፡ እንዲሁም በፈለጉት ጊዜ ውይ ይቱን ማቋረጥ ይችላሉ ፡፡ በለመሳተፍዎ ወይም ውይይቱን በማቋረጥዎ በእርስዎም ሆነ በማንኛዉም የቤተሰብዎ አባል ላይ የሚደርስ ተጽእኖ አይኖርም፡፡ ከእርስዎ ትክክለኛ እና ታማኝ መረጃ እፈልጋለሁ ፡፡ ምክንያቱም የሚሰጡን መረጃ ለትክክለኛ ምክረ ሀሳብ እና ድምዳሜ ስለ ሚጠቅም እና ለፌዴራል ጤና ጥበቃ ሚኒስቴር እና ጤና ተቋማት ለህብረተሰቡ የሚሰጡትን አገልግሎቶች እንዲያሻሽሉ ስለሚረዳ ነዉ ። መቀጠልእችላለሁ ?

1. አዎ ቀጥል/ይ ☐

2. አልተስማማሁም አመስግ ነህ/ሽ ጨርስ/ሺ ☐

ተጨማሪ ጥያቄ ካለዎት በማንኛዉም ጊዜ ከዚህ በታች በተጠቀሰዉ

አድራሻ ዋና ተመራማሪዉን ማግኘት ይቻላል፡፡

ታደሰ አያና ዋና ተመራማሪ

Tel: +251-920-77-48-21, Email: tadesseay6@gmail.com

አባሪVII: የአማርኛ ቅጅ የፈቃደኝነት ማረጋገጫ ቅፅ

እኔ በጥናቱ ላይ እንድሳተፍ የተጠየቅኩ ከዚህ በላይ በጥናቱ መረጃ ቅፅ ላይ የተቀመጠዉን ነገር በአግባቡ በመረዳት ከእኔ የሚጠበቀዉን ሁሉ አዉቄያለሁ፡፡ ከዚህም ሌላ በጥናቱ ላይ ተሳታፊ ብሆን እኔ የምሰጣቸዉ መረጃዎች ለሶስተኛ አካል ተላልፈዉ እንደማይሰጡ እና ስሜም እንደማይካተት ተረድቻለሁ፡፡ በተጨማሪም ዉይይቱን የማልፈልገዉ ከሆነ በማንኛዉም ሰዓት ያለምንም ምክንያት ማቆም እንደምችል እና በማቆሜም እኔ ወይም ቤተሰቦቼ ከድርጅቱ በሚያገኙት አገልግሎት ላይ ምንም ዓይነት ተፅዕኖ እንደማይኖረዉ አዉቄያለሁ፡፡

በቃለ መጠይቁ ለመሳተፍ ፈቃደኛ ነዎት?

1. አዎ ቀጥል/ይ ☐

2. አልተስማማሁም አመስግነህ/ሽ ጨርስ/ሺ ☐

የቃለመጠይቅ አድራጊዉ/ዋ ስምምነት

ተሳታፊዋ በጥናቱ ላይ ለመሳተፍ ፈቃደኝነቷን የሚገልፅ ስምምነት በፅሑፍ መልክ መዉሰዴን እና ስምምነቱም ትክክለኛ መሆኑን አረጋግጣለሁ፡፡

የቃለ መጠይቅ አድራጊዉ/ዋ ስም _________________ፊርማ _______ቀን ---- /----/2016ዓ.ም

የተቆጣጣሪዉ/ዋ ስም _____________________ፊርማ _________ቀን -----/-----/2016 ዓ.ም

አባሪVIII: የአማርኛ ቅጅ መጠይቅ

መለያ ኮድ ቁጥር_____________

በጤና ተቋማት የሚሰጠዉን አክብሮት የጎደለዉ እና እንግልት የበዛበት የወሊድ አገልግሎት እ ና ተያያዥ ጉዳዮችን ለማጥናት የተዘጋጀ መጠይቅ

**ክፍል አንድ፡ማህበራዊ እና ዲሞግራፊያዊ ሁኔታዎች ከዚህ በታች ያሉ ጥያቄዎችን በአግባቡ ከተረዱ በኋላ ምላሽ ይስጡ**				
**ተ .ቁ**	**ጥያቄ**	**መልስ**	**እለፊ/ፍ**	**ኮ ድ**
101	እድሜዎ ስንት ነ ዉ	1. ------------ ዓመት		
102	በአሁኑ ሰዓት የጋብቻ ሁኔታዎ ምን ይመስላል ?	1. ያላገባች<br>2. ያገባች<br>3. የፈታች<br>4. የሞተባት		
103	የመኖሪያ አካባቢዎ የት ነ ው ?	1. ከተማ<br>2. ገጠር		
104	የየተኛዉ ሃይማኖት ተከታይ ነዎት?	1. ኦርቶዶክስ<br>2. ሙስሊም<br>3. ፕሮቴስታንት<br>4. ካቶሊክ<br>5. ሌላ (ይገለጽ)-----------		
105	ከፍተኛ የትምህርት ደረጃሽ?	1. ማንበብና መፃፍ የማትችል<br>2. ማንበብና መፃፍ ብቻ የምትችል<br>3. ከ1ኛ-8ኛ የተማረች<br>4. ከ9ኛ-12ኛየተማረች		

		5. ኮሌጅና ከዚያ በላይ		
106	በአሁኑ ሰዓት እርሰዎ የሚሰሩት ስራ ምንድን ነው?	1. የመንግስት ሰራተኛ<br>2. ነጋዴ<br>3. የግል ተቀጣሪ<br>4. ተማሪ<br>5. የቤት እመቤት<br>6. ሌላ (ይገለጽ)----------		
**ክፍል ሁለት ፡ የእናትየዋ የወሊድ ታሪክ**				
**ተ .ቁ**	**ጥያቄ**	**መልስ**	**እለፊ/ፍ**	**ኮ ድ**
201	ለአሁኑ እርግዝናዎ የቅድመ ወሊድ አገልግሎት ክትትል<br>አድርገዋል ?	1. አ ዎ<br>2. የለም	አላደረኩ ም ከሆነ ወደ ጥያቄ 203 እለፊ/ፍ	
202	በዚህ እርግዝና ወቅት ምን ያህል ጊዜ የቅድመ ወሊድ ክትትል አድርገው ነበር?	1. ---------በቁጥር		
203	የእርግዝና ሁኔታን የታቀደ እና የተፈለገ ነበር?	1. አ ዎ<br>2. አደለም		
204	ይህ ስንተኛ ወሊድዎት ነው ?	1. ---------በቁጥር		
205	የአሁኑን ልጅዎን በምንድን ነው የወለዱት ?	1. በምጥ<br>2. በመሳሪያ ታግዤ<br>3. በቀዶ ጥገና		
206	በወሊድ ወቅት ችግር አጋጥሞዎት ነበር?	1. አዎ<br>2. የለም	የለም ከሆነ ወደ ጥያቄ 208እለፊ /ፍ	
207	ለተ.ቁ 206 መልሰዎ አዎ ከሆነ ያጋጠመዎት ችግር ምንድን ነው? (የወሊድ ካርዱን ይመልከቱ)	1. የደም መፉሰስ<br>2. የደም ግፊት		

		3. የማህጸን መሰንጠቅ<br>4. ሌላ (ይጥቀሱ)------------		
208	ህፃኑ በምጥ ጊዜ/ከተወለደ በኋላ ያጋጠመው ችግር ነበር?	1. አዎ<br>2. የለም	የለም ከሆነ ወደ ጥያቄ 210 እለፊ/ፍ	
209	ለተ.ቁ 208 መልሰዎ አዎ ከሆነ ያጋጠመው ችግር ምንድን ነበር? (የወሊድ ካርዷን ይመልከቱ)	1. መታፈን<br>2. ትክክለኛ ያልሆነ የፅንስ አቀማመጥ<br>3. የጨቅላ ህፃኑ ላይ አካላዊ ጉዳት መድረስ<br>4. ሌላ (ይጥቀሱ)-----------		
210	የወለድሽው በምን ሰዓት ነበር ?	1. ቀን<br>2. ማታ		
**ክፍል ሶስት፡ የተገልጋይ የጤና አገልግሎት ሁኔታ መረጃ**				
**ተ .ቁ**	**ጥያቄ**	**መልስ**	**እለፊ/ፍ**	**ኮድ**
301	ምጥ ላይ በነበርሽበት ጊዜ ያንቸ ሰው/ረዳት አብሮሽ ነበር?	1. አዎ<br>2. አይደለም		
302	ከዚህ በፊት እዚሁ ጤና ተቋም ወልደሽ ታዉቂያለሽ?	1. አዎ<br>2. አይደለም		
303	ከዚህ በፊት እዚሁ ጤና ተቋም ከወሊድ ውጭ ተገልግለሽ ታዉቂያለሽ?	1. አዎ<br>2. አይደለም		
304	አገልግሎቱን እዚህ እንዲያገኙ ከሌላ የጤና ተቋም ሪፈር ተብለው/ተፅፎለዎት ነው?	1. አዎ<br>2. አይደለም		
305	ከወለዱ በኋላ ስንት ቀን በጤና ተቋም ቆዩ?	1 -------በቀን		
306	በቅድመ ወሊድ ወይም ወሊድ ሰዓት የኤች አይቪ ምርመራ ተደርጎልሻል? (የወሊድ ካርዱን ይመልከቱ)	1. አዎ<br>2. አይደለም	አዎ ከሆነ ወደ ጥያቄ 307 እለፊ/ፍ	

307	ለተ.ቁ 306 መልሰዎ አዎ ከሆነ ውጤቱ ምንድን ነበር? (የወሊድ ካርዱን ይመልከቱ)	1. አለባት<br>2. የለባትም<br>3. የተሳሳተ ውጤት		
**ክፍል አራት አጠቃላይ የጤና ተቋም እ ና የባለሙያዉ ተያያዥ መረጃ**				
**ተ .ቁ**	**ጥያቄ**	**መልስ**	**እለፊ/ፍ**	**ኮ ድ**
401	የቅድመ ወሊድ አገልግሎት ክትትል የት ነበር ያደረግሽዉ?	1. ጤና ጣብያ<br>2. ሆስፒታል<br>3. የግል ክሊኒክ		
402	አሁን የወለድሽበት የጤና ተቋም ?	1. ጤና ጣብያ<br>2. ሆስፒታል		
403	ያዋለደሽ የጤና ባሙያ ፆታው ምንድን ነበር?	1. ወንድ<br>2. ሴት		
404	ያዋለዳት የጤና ባለሙያ የሰለጠነበት የሙያ ዘርፍ? (የወሊድ ካርዷን ይመልከቱ	1. አ.ኤ.ስ.ኦ<br>2. ሚድዋይፈሪ<br>3. ጤና መኮነን<br>4. ዶክተር<br>5. ሜዲካል ኢንተርን<br>6. ሌላ (ይጥቀሱ)-------		
**ክፍል አምስት ፡ አክብሮት የጎደለዉ እና እንግልት የበዛበት የወሊድ አገልግሎት ቀጥሎ የተዘረዘሩት ጥያቄዎች በጤና ተቋሙ በምጥ እና ወሊድ ወቅት ስለአጋጠሙሽ ሁኔታዎች ይመለከታሉ፡፡ ለእያንዳንዱ ጥያቄ ያጋጠሙሽን ኩነቶች በማስታወስ ምላሽ ትሰጪአ ለሽ**				
**ተ .ቁ**	**ጥያቄ**	**መልስ**	**እለፊ/ፍ**	**ኮ ድ**
**ወላድ እናቶች ከአካላዊ ጉዳት እና እንግልት የመጠበቅ መብት**				
501	በምጥ እና በወሊድ ጊዜ በጤና ባለሙያ ው/ዋ አካላዊ ጉዳት ደረሰበዎት ነበር ? (ሃይል መጠቀም ፣ መደብደብ ፣ ማጋጨት ፣መገፍተር ?	1. አዎ<br>2. አይደለም		
502	ጤና ባለሙያው/ዋ ትእዛዛቸውን እንድታከብሪ አስፈራርተዉሽ ነበር ?	1. አ ዎ<br>2. አይደለም		

503	ጤና ባለሙያው/ዋ ማደንዘዣ ሳይጠቀሙ ማህጸሽን ጠግነዉ ነበር?	1. አዎ<br>2. አይደለም<br>3.ተፈፃሚየማይሆን		
504	በምጥና በወሊድ ጊዜ በጤና ባለሙያው/ዋ ከአልጋዉ ጋር አካሊዊ ዉጥረት ደረሰበዎት ነበር ?	1. አዎ<br>2. አይደለም		
505	ባለሙያዎች በምጥ እና ወሊድ ወቅት በምትፈልጊዉ በኩል የመተኛት ፍላጎትዎን አላከበሩም ነበር ?	1. አዎ<br>2. አይደለም		
506	በምጥ ወቅት የጤና ባለሙያዎች የመንቀሳቀስ ነጻነት አልሰጠዎትም ነበር?	1. አዎ<br>2. አይደለም		
507	ጤና ባለሙያው/ዋ ህፃኑን ለማዋለድ ሆድዎን ወደታች መጫን ነበር?	1. አዎ<br>2. አይደለም<br>3.ተፈፃሚየማይሆን		
508	ጤና ባለሙያ ው/ዋ ያለህክምና ትዕዛዝ ምግብ ወይም ፈሳሽ እንዳትወስጂ ከልክለለዉሽ ነበር ?	1. አዎ<br>2. አይደለም		
**ወላድ እናቶች ትክክለኛ መረጃ የማግኘት ፣ የመወሰን፣ የመፍቀድ ፣የሚመቻቸዉን የመምረጥ መብት**				
509	ጤና ባለሙያው/ዋ እራሱን/ሷን አስተዋውቆ/ቃ እና ሰላምታ ለእርስዎ እና አብሮወት ላለ ሰው አልሰጡም ነበር ?	1. አዎ<br>2. አይደለም		
510	ጤና ባለሙያዎች የምጥሽን ሁኔታ በየጊዜዉ አያስረድዎትም ነበር ?	1. አዎ<br>2. አይደለም		
511	ጤና ባለሙያዎች ጥያቄ እንድትጠይቂ አላበረታቱዎትም ነበር ?	1. አዎ<br>2. አይደለም		
512	ጤና ባለሙያ ው/ዋ በምጥ ሰዓት ምን እየተሰራ እንደሆነ እና ምን እንደሚያጋጥም አልነገሩዎትም ነበር ?	1. አዎ<br>2. አይደለም		
513	ጤና ባለሙያዎች ከማንኛዉም ምርመራ በፊት የአንቺን ፈቃደኝነት አይጠይቁም ነበር ?	1. አዎ<br>2. አይደለም		
514	ጤና ባለሙያ ው/ዋ በቀዶ ጥግና እንድትወልጅ አስገድደዉሽ ነበር?	1. አዎ<br>2. አይደለም		
**ወላድ እናቶች አገልግሎት በሚያገኙበት ጊዜ ምስጥራዊነቱ የጠበቀ መሆን መብት**				
515	ጤና ባለሙያው/ዋ የአንቺን ሚስጢር ለመጠበቅ በምርመራውቅ ት መጋረጃ አልተጠቀሙም ነበር ?	1. አዎ<br>2. አይደለም		
516	በወሊድ ወቅት ክፍል ውስጥ ጤና ባለሙያው/ዋ ሌላ ሰው እንዲገባ አድርገው ነበር?	1. አዎ<br>2. አይደለም		

517	ጤና ባለሙያው/ዋ የእርስዎን ሚስጥራዊ መረጃ ሌሎች በሚሰሙት ሁኔታ ተዎያይተዉ ነበር ?	1. አዎ<br>2. አይደለም		
**ወላድ እናቶች ክብር የጠበቀ አገልግሎት የማግኘት መብት**				
518	ባለሙያዎች በአክብሮት አላናገሩሽም ነበር ?	1. አዎ<br>2. አይደለም		
519	ባለሙያዎች አስፈራርተዉሽ ወይም ሰድበዉሽ ነበር?	1. አዎ<br>2. አይደለም		
520	ጤና ባለሙያው እርሶን በሚመለከት መጥፎ አስተያት ሰጠዉ ነበር	1. አዎ<br>2. አይደለም		
521	በምጥ ህመም ወቅት ጤና ባለሙያው/ዋ ጮህዉበት ወይም ገፍትረዉዎት ነበር	1. አዎ<br>2. አይደለም		
522	ጤና ባለሙያው/ዋ ጓደኛዉ ወደ ማዋለጃ ክፍል እንዲገባ አድርገዉ ነበር?	1. አዎ<br>2. አይደለም		
**ወላድ እናቶች እኩል እና አድልኦ የሌለዉ አገልግሎት የማግኘት መብት**				
523	ጤና ባለሙያው/ዋ በዘር ፣ በብሄር ፣ በትምህርት ደረጃ ወይም በኢኮኖሚ ሁኔታ ምክንያት አድልኦ እና ማግለል ነበር ?	1. አዎ<br>2. አይደለም		
524	ጤና ባለሙያው በእዴሜ (ከ18 ዓመት በተች በመሆንሽ)<br>ወይም በእድሜሽ መግፋት ምክንያት አድሎ ነበር?	1. አዎ<br>2. አይደለም		
525	ጤና ባለሙያው/ዋ ኤች. አይ. ቪ ስላለብሽ ብቻ አድሎ ነበር ?	1. አ ዎ<br>2. አይደለም<br>3.ተፈፃሚየማይሆን		
**ወላድ እናቶች ትኩረት እና ክትትል ሳይደረግላቸዉ መቆየት የለባቸዉም**				
526	ጤና ባለሙያዎች ብቻሽን ወይም እንክብካቤ ሳታገኚ ትተዉሽ ነበር ?	1. አዎ<br>2. አይደለም		
527	ምጥ ላይ እያሉ ልጁ በሚወጣበት ግዜ ጤና ባለሙያው ትተዎት ሂደዉ ነበር?	1. አዎ<br>2. አይደለም		
528	ባለሙያዎች በምትፈልጊያቸዉ ጊዜ በፍጥነት አይመጡም ነበር ?	1. አዎ<br>2. አይደለም		
**ወላድ እናቶች ከፍላጎታቸዉ ዉጪ መዘግት እና መቆየት የለባቸዉም**				
529	ከሆስፒታሉ የሚወጡበት ግዜ ሂሳብ ባለመክፈልዎ ምክንያት ዘግይቶ ነበር ?	1. አዎ<br>2. አይደለም		
530	ያለፍላጎትዎት በጤና ተቋም እንዲቆዩ ተደርገው ነበር ?	1. አዎ<br>2. አይደለም		

አባሪ IX: የአማርኛ ቅጅ ለቁልፍ መረጃ ሰጭ መረጃ እናየፈቃደኝነት ማረጋገጫ ቅፅ

እነደምን አደሩ/ዋሉ ;እንደምንነዎት; ስሜ_________________________ እባላለሁ፤በጎነንደር ዩኒቨርሲቲ ዉስጥ ለሚካሄድ ጥናትና ምርምር የጥናቱ መረጃ ሰብሳቢ ነኝ።የዚህ ጥናት ዋና ዓላማ የእናቶችን በአክብሮት የወሊድ አገልግሎት የመሰጠት እንዲሁም ተያያዥ ጉዳዮችን ለማጥናት የተመረጡ በደብረታቦር ከተማ ውስጥ የሚገኙ የጤና ተቋማትን ለመዳሰስ ነው። እርስዎ በጥናቱ እንዲሳተፉ የተመረጡት ለጥናቱ ዋና አላማ ስኬታማነት የተሻለ መረጃ ይኖረወታል ተብሎ ታስቦ ሲሆን ቃለ ምልልሱ በግምት 30 ደቂቃ ይፈጃል። እርስዎ የሚሰጡኝ መረጃ ሚስጥራዊነቱ ሙሉ በሙሉ የተጠበቀ ነው።መረጃዉ ለጥናቱ ዓላማ ብቻ ይውላል። ስም አይመዘገብም። ዉይይቱ የመሚቀረጸዉ በእርስዎፈድቃ ብቻ ነዉ፤ የድምፅ ዉሂቡን ወደጽሑፋዊ መልክ ከገለበጥን በኍላ የተቀዳዉ መረጃ ይሰረዛል ፤በጥናቱ የመሳተፍም ሆነ ያለመሳተፍ መብት ያለዎት ሲሆን በመሀልም ጥያቄዎችን የማቋረጥ መብትዎ የተጠበቀ ነው። ነገር ግን እርስዎ የሚሰጡን እውነተኛ መልስ እናቶችን በአክብሮት የወሊድ አገልግሎት አሰጣጥና እንዲሁም ተያያዥ ጉዳዮች ላይ ችግሮችን ለመለየት ይረዳል።

በቃለ መጠይቁ ላይ ለመሳተፍ ፈቃደኛ ነዎት?

እስማማለሁ ☐ አልስማማም ☐

ጠያቂ፡ ስም ____ፊርማ _____________

አባሪX: ለቁልፍ መረጃ ሰጪ ጤና ባለሙያዎች የተዘጋጀ ጥልቅ ቃለ መጠይቅ መምሪያ

የተሳታፊዎች ማህበራዊ እና ዲሞግራፊያዊ ሁኔታዎች

መለያ ቁጥር-------------

ተ .ቁ	ሁኔታዎች	
1	እድሜ	
2	የትምህርት ደረጃ	
3	የስራ ልምድ አሁን ባለው የስራ መደብ	
4	ሃላፊነት አሁን ባለው የስራ መደብ	

1. አክብሮት የተሞላበት የእናቶች እንክብካቤ ለእርስዎ ምን ማለት እንደሆነ ሀሳብዎን ያጋሩን? በእርስዎ አስተያየት፣ በዚህ የጤና ተቋም ውስጥ የሚሰጠውን አክብሮት የተሞላበት አገልግሎት ጥራት እንዴት ያዩታል? ከመልስዎ ጀርባ ያለውን ምክንያት ቢያብራሩልኝ?
2. አክብሮት የተሞላበት የእናቶች እንክብካቤ እንደሰጠህ/ሽ እራስህን/ሽ አግኝተህ/ሽ ታውቃለህ/ሽ? ከሆነስ መቼ እና እንዴት ?
3. አክብሮት የተሞላበት የእናቶች እንክብካቤ አሰጣት ላይ ተጽእኖ የሚያደርጉ ቁልፍ ነገሮች ምንድን ናቸው ብለው ያምናሉ? ለምን እና እንዴት ?
4. ከእርስዎ እይታ አንፃር፣ አክብሮት የተሞላበት የእናቶች እንክብካቤ አሰጣት በበቂ ሁኔታ እየተሰጠ ይመስልዎታል? እንዴት ?
5. በመጨረሻም፣ በርዕሱ ዙሪያ መወያየት አለብን የሚሉት ተጨማሪ ሃሳቦች አሉዎት?

ስለ ጊዜዎ እና ተሳትፎዎ እናመሰግናለን!!
