# Supplementary material for: Respectful maternity care and associated factors among mothers who gave birth at public health institutions in Debre Tabor town, Northwest Ethiopia: a mixed-methods study
Source: Front Glob Womens Health. 2025 Jan 23;6:1513906. doi: 10.3389/fgwh.2025.1513906 (PMC11798984; doi:10.3389/fgwh.2025.1513906)
Supplement: Supplementary file 2 [file Datasheet2.pdf]

Type of data: Qual

የቃለ መጠይቁ ቀን: 24/04/2016

የተሳታፊ: KII 1

የቃለ መጠይቁ ቆይታ: 07:06 ሰከንድ

ጠያቂ: አክብሮት የተሞላበት የእናቶች እንክብካቤ ለእርስዎ ምን ማለት እንደሆነ ሀሳብዎን ያጋሩን? በእርስዎ አስተያየት፣ በዚህ የጤና ተቋም ውስጥ የሚሰጠውን አክብሮት የተሞላበት አገልግሎት ጥራት እንዴት ያዩታል? ከመልስዎ ጀርባ ያለውን ምክንያት ቢያብራሩልኝ?

ተሳታፊ: በኛ ሆስፒተል ሲ አር ሲ አገልግሎት በተወሰነ መልኩ አለ።

ጠያቂ: አክብሮት የተሞላበት የእናቶች እንክብካቤ እንደሰጠህ/ሽ እራስህን/ሽ አግኝተህ/ሽ ታውቃለህ/ሽ? ከሆነስ መቼ እና እንዴት ?

ተሳታፊ:ሙሉ ለሙሉ ለማድረግ የሚያግዱ ምክኒያቶች፤ ኢፍራእስትራክቸር ስንል እስክሪን የለም ፔሼንቶች ፕራይቤሲ ለመጠበቅ፤ ጥበቃው በጣም የተዳከመ ነው። አንድ እናት ልትወልድ ስትመጣ የሰፈር ሰው እቤተሰብ ተሰብስቦ ነው ሚመጣ እነሱን አንተ ነህ ምታስወጣ። ኢብን ፍዚካል ኤግዛሚኔሽን እየሰራህ ዘለው ይገቡብሐል ተሰብስበው። የባለሙያ እና የታካሚዎች ቁጥር አለመመጣጠን፤ ሌላው ደግሞ ወላዶች የባለሙያውን ምክር አልሰማ ሲሉ፤ሌላው ደግሞ አብዛኛው ጊዜ ሌሊት ነዉ ወላድ ሚበዛው በኛ ሆስፒታል ሌሊት ላይ የባለሙያ ቁጥሩ ይቀንሳል፤ የእናቶች ቁጥር ይጨምራል ፤በዚያ ላይ ሌሊት ላይ ድካም አለ በድካም ሰዓትት መሰልቸት ይኖራል ፤ በዚህም ምክኒያት እናቶች ሚፈልጉትን የተሟላ ሲ አር ሲ ላይሰጣቸው ይችላል፤ ሌለኛው ደግሞ ባለሙያን ፐሮሞት አለማድረግ፤ ለባለሙያው የሚገባውን ክብር አለመስጠት፤ እኔ ወደ ስምንት አመት አገልግያለሁ ፕሮሞት አልተደረግሑም የትምህርት እድል የለም ይህን ያህል አገልግየ ስምንት ሽ ብር ነው ደሞዜ ፤ ይህ በኑሮ እና በስራ አለመርካት ፤ ለምሳሌ በኔ ተመሳሳይ እድሜ አገልግሎት ያላቸው ሌላ ሙያላይ ያሉ ሰዎች ከነዚያ ጋር እያወዳደርኩኝ ያለመርካት ሁኔታ ስለሚኖር ፤ ቀስ በቀስ ለሙይያው ፍቅሩ እየቀነሰ ይመጣል ፤በዚያ ምክኒያት ለእናቶች የምናደርገውን አገልግሎት በተወሰነ እንዲቀንስ ያደርገዋል።

ጠያቂ:በአንተ አስተሳሰብ አንዲት እናት በምትወልድበት ሰዓት በአክብሮት አገልግሎቱን እንድታገኝ/እንዳታገኝ ሊያደርጉ የሚችሉ ተያያዥ ጉዳዮች ምን ምን ሊሆኑ ይችላሉ ብለህ ታምናለህ? እንዴት?

ተሳታፊ:አንዳንድ ጊዜ የህፃን መታፈን ሲኖር ደም መድማት ሲኖር ያነን ማኔጅ ለማድረግ ላይሰሙ ይችላሉ፤ ባለሙያው ህፃኑን ማትረፍ እንዳይታፈን እናቲቱን ደም እንዳይፈሳት ቅድሚያ የምሰጠው እሱን ስለሆነ በዚያ ተባባሪ አልሆንብለው ሲቀሩ፤

ጠያቂ: አንተ በቅርብ አለቃህ/በስራ ባልደረባህ የክብር መነፈግ ቢደርስብህ ምን ይሰማሀል? ለምን?

ተሳታፊ:በኛሆስፒታል ሁኔታ እንደሚድዋይፍ በአስተዳደራዊ ሑኔታዎች ተገፍተናል ብለን እናስባለን ፤ የምንጠይቃቸው ጥያቄዎች ፖዘቲብ መልስ አይሰጠነም ፤በዚህ ምክኒያት ሙያውን እንድንጠላው ያደርገናል፤ በቅርብ አለቃ የሙብት ጥያቄዎች መልስ አይሰጠነም እነዚህ ነገሮች ተዕኖ አላቸው፤

ጠያቂ:የወሊድ አገልግሎቱን የሚሰጠው/ባለሙያው አንዲት እናት በምትወልድበት ሰዓት ስነ-ምግባር በጎደለው ሰብዕና ሲያስተናግድ ተይዞ ተጠያቂ የሚሆነው እንዴት ነው?

ተሳታፊ:በ ሲ አር ሲ እስካሁን ድረስ ተጠያቂ ሲደረግ አላየሁም፤ የሙብት ጥሰት ቢያደርሱም ቢሳደቡም ሰበዊ ነት ካላዛእዉ በስተቀር በድስብሊን ወይም ተጠያቂነት ሚባለውን ነገር አላየሁም የሆነ ባስ ያለነገር ካልሆነ እንደ እስቲል በርዝ አይ ዩ ፍ ዲ ካልሆነ በቀር፤ በዚህ በ ሲ አር ሲ ጉዳይ ተሳደበ ወይም መብቷን ጣሰ ተብሎ ሰው ሲጠየቅ አላየሁም ይሄ እንደ እንድ ምክኒያት ሊሆን ይችላል፤

ጠያቂ:ሳናነሳው የቀረ ነገር ግን ለጥናቱ ይጠቅማል የምትለው/ይዉ ሀሳብ ካለ የመጨረሻ እድል ልስጥህ/ሽ?

ተሳታፊ: ባለሙያው ቢጠየቅና ያለው ክፍተቱ ቢጠና እነዚህ ነገሮች ስኖሩ ተጠያቂነት ቢኖር፤ በሆስፒታሉ የፔሽንት እና የባለሙያ ቁጥር እስታንዳርድ ቢወጣለት ድካም ይቀንሳል፤ ሌላው በቅርብ ሓላፊ የሚነሱ ነገሮች ቢፈቱ ፤የትምህርት እድል ቢኖር ፤ጥቅማጥቅም ቢኖር፤ ሙያውም እነዚህ ነገሮችም ሊስተካከሉ የሚችሉት ባለሙያው ሙያውን እንዲወደው በማድረግነው.።

Type of data: Qual

የቃለ መጠይቁ ቀን:24/04/2016

የተሳታፊ: KII 2

የቃለ መጠይቁ ቆይታ:11:31 ሰከንድ

ጠያቂ:አክብሮት የተሞላበት የእናቶች እንክብካቤ ለእርስዎ ምን ማለት እንደሆነ ሀሳብዎን ያጋሩን? በእርስዎ አስተያየት፣ በዚህ የጤና ተቋም ውስጥ የሚሰጠውን አክብሮት የተሞላበት አገልግሎት ጥራት እንዴት ያዩታል? ከመልስዎ ጀርባ ያለውን ምክንያት ቢያብራሩልኝ?

ተሳታፊ: ሲ አር ሲ በተመለከተ እኛጋ ሚመጡ ሁሉም እናቶች አክብሮት በተሞላበት የጤና አገልግሎት ነው የሚያገኙት። ያም ሲባል እነሱ በሚፈልጉት ዎይ ሚፈልጉትን አገልግሎት ያገኛሉ ተብሎ ይታሰባል፤ በኛ ሆስፒታል ዉስጥ፤

ጠያቂ: አክብሮት የተሞላበት የእናቶች እንክብካቤ እንደሰጠህ/ሽ እራስህን/ሽ አግኝተህ/ሽ ታውቃለህ/ሽ? ከሆነስ መቼ እና እንዴት ?

ተሳታፊ: የኬዝ መብዛት እና የባለሙያ ቁጥር አለመመጣጠን ሊኖር ይ ችላል ፤ ለምሳሌ ሁለት ሶስት እናት አንዴ ፎሎው ልታደርግ ትችላለህ ፤ በአንዴ ሁለት ሶስት እናት ፎሎው ስታደርግ እንደዚህ አይነት ነገሮች ሊጋጥሙ ይችላሉ ፤ከዚህ አንጻርም እናትየዋ ተገቢዉን እንክብካቤ ማታገኝበት አጋጣሚ ሊኖር ይችላል፤ የድካም ስሜት ሊኖር ይችላል ፤ ናይት ከሆነ ደግሞ የእንቅልፍ ሰዓት ስለሆነ የድካም ስሜት ይኖራል፤ አንዳንዴ የማይመች ሰዓትም ሊኖር ይችላል፤ ይሄ ይሄ ነገር ሲደማመር፤ ሲ አር ሲዉ ላይተገበር ይችላል፤ አንዳንዴ አንድ እናት ልትወልድ ስትመጣ አስር አቴንዳንት አብሮ ሊመጣ ይችላል ፤ ብራይቤሲ ለመጠበቅ ሲባል ያንን አስታማሚ አብሮ የመጣን ልታስዎጣ ትችላለህ፤ ያሰዉ ደግሞ አልዎጣም ሊል ይችላል፤ የጥበቃ ሰራተኛ እጥረት አለ በዚህ ዎይ ሲአር ሲ በተሞላበት መልኩ ላታስተናግድ ትችላለህ፤ የሚመጣዉ አስታማሚ ከሚመጣዉ እናት ጋር ሲደመር የጥበቃ ሁኔታው ሲአርሲዉን ወይም እናቶችን ተገቢዉን እንክብካቤ ሳያገኙ የሚሄዱበት ሁኔታ ይኖራል፤ ይሄ ሁሉ የሚሆነው ባለሙያዉ የሚያገኘዉ ነገር ኑሮ አደለም ለእናቶች ሲባል፤ እናቶች ህጻናቸዉን አቅፈዉ እንዲሄዱ ሲባል ነው፤

ጠያቂ: በአንተ አስተሳሰብ አንዲት እናት በምትወልድበት ሰዓት በአክብሮት አገልግሎቱን እንድታገኝ/እንዳታገኝ ሊያደርጉ የሚችሉ ተያያዥ ጉዳዮች ምን ምን ሊሆኑ ይችላሉ ብለህ ታምናለህ? እንዴት?

ተሳታፊ: አንዳንዴ ሲ አር ሲን እነሱ ባሰቡት ልክ ላያገኙ ይችላሉ፤ ምክኒያቱም አሁን ለምሳሌ የህፃን መታፈን ወይም ፌታል ዲስትረስ የሚባለው ነገር ቢከሰት እና ህፃኑን ለማዳመጥ ብዙ ጊዜ በጀርባሽ ተኚ ምንላቸዉ እናቶች አሉ፤ ያን ደግሞ ላይተገብሩልን ይችላሉ ፤የፖዚሽን ወይም የአተኛኘት ሁኔታዉ የማይመች ስለሆነ፤ እነሱ ያኔ በጀርባችን አንተኛም የማለት ነገር ሊያመጡ ይችላሉ፤ ያበሚሆን ሰዓት ህፃን ከሚታፈን እና ህፃኗን ከምታጣ የእናትዮዋን መብትልንጋፋ እንችላለን ፤ ስለዚህ በዛ ሰዓት ይች እናት ልጇን እንዳታጣ በየ ደቂቃዉ በየሰዓቱ መደመጥም መታየትም ስላለበትህፃኑ፤ በዚህ ሁኔታ ሲ አር ሲዉ ላንተገብር እንችላለን ፤ ሲ አር ሲዉ ምታገኝበት ሁኔታ ባለሙያዉ የሚላትን ማንኛዉንም ነገር መተግበር አለባት ፤ ምክኒያቱም ባለሙያው የተቀመጠዉ ህፃኗን እንዳታጣ እሷም ደና እንድትሆን ነዉ ፤ ነገርግንሲ አር ሲዉ ማታገኝበት ምክኒያት አንዳንዴ ባለሙያ የሚላትን ነገር ላትተግብር ትችላለች፤ ለምሳሌ በግራ ጎንሽ ተኚ ተብላ በቅኝ ጎን ምትተኛ ከሆነ፤ አንዳንዴ የልጇ መታፈን ሁኔታ ሊያመጣ ይችላል ፤ ስለዚህ እንዚህ ነገሮች የማትተገብር ከሆነ ሲ አር ሲዉ ላይተገበር ይችላል ማለት ነዉ፤ ምክኒያቱም ደግሞ ለባለሙያዉ ምንም የሚሰጠዉ ጥቅም አይኖርም ሚሰጠዉ ጥቅም ለሷ ነው፤ ህፃኗ ሊታፈንባት ይችላል ፤ህፃኗን ለማትረፍ ፤ እሷንም ለማትረፍ ስለሆነ አንዳንድ ነገሮች ላይ ሲ አር ሲዉ ላይተገበር ይችላል ፤ ሌላ ሰትዮዋ ለመዉለድ ፖዚሽን ላትይዝ ትችላለች፤ በዚህ ሁኔታ ሲ አር ሲዉ ላይተገበር ይችላል፤ እኔ ሲ አር ሲ አልተገበርኩም ብየ የማስበው የህፃን መታፈን ሲኖር በየ አስራምስት ደቂቃዉ በየአምስትደቂቃዉ በየአስር ደቂቃዉ የህፃን የልብ ምት መደመጥ አለበት፤ ስለዚህ ያኔ በየአምስት ደቂቃዉ በየአስር ደቂቃዉ እሷን ልናስቸግራት እንችላለን፤ ብዛ ሰዓት የምንፈልገዉን ነገር ላትተገብርልን ትችላለች፤ በዚህ ሁኔታ እሷ ካልተገበረች እኛ ደግሞ የእሷን ህፃን ለማትረፍ ስለሆነ ሲ አር ሲዉ ላይተገበር ይችላል፤ ባለሙያው እነሱን ለማገዝ ስለሆነ ባለሙያው የሚላትን ማድረግ አለባት ፤ ምክኒያቱም ማንኛዉም ባለሙያ እዚህ ያለዉ እናትን እና ልጅን ለማገዝ ነው፤ ስለዚህ እናቶች ሊረዱን ይገባል፤

ጠያቂ:የወሊድ አገልግሎት የሚሰጠውን ባለሙያ የስራ የቅርብ አለቃው ባያከብረው በወላድ እናቷ ላይ ምን ያስከትላል ብለህ ታስባለህ? እንዴት?

ተሳታፊ: ስራ ለሰዉ ብለህ አደለም የምትሰራው ስራ የምትሰራዉ ለራስህ ነው፤ አለቃህ ስራህ ነዉ፤ ስለዚህ በቅርብ አለቃህ ምንም ነገር ቢደረሰብህ የመደበር ስሜት ሊኖር ይችላል፤ በዚህ ሁኔታ ለእናቶች ተገቢዉን እንክብካቤ ተገቢዉን ክብር ላትሰጥ ትችላለህ፤ እናቶችን የመጨቃጨቅ ተገቢዉን ሲአርሲ የተሞላበት አገልግሎት ላትሰጥ ትችላለህ ብየ አስባለሁ፤

ጠያቂ:የወሊድ አገልግሎቱን የሚሰጠው/ባለሙያው አንዷት እናት በምትወልድበት ሰዓት ስነ-ምግባር በጎደለው ሰብዕና ሲያስተናግድ ተይዞ ተጠያቂ የሚሆነው እንዴት ነው?

ተሳታፊ: ስለዚህ አልፎ ተርፎ ተጠያቂነት ሊኖር ይችላል፤ ግን ተጠያቂነት ጥሩ ነው ብየ አላስብም ምክኒያቱም ባለሙያው ሲአርሲ ላይተገብር የሚችለው አገልግሎቱን ለሚያገኘዉሰው ብሎ ነው ፤ ባለሙያው ሁሉ ነገር የሚያደርገው የሚያገኘዉ ነገር ኑሮ አደለም፤ መጨረሻላይ ሰላም ከሆነ ባለሙያዉ ከዚህ ከዚህ አንጻር ነው ብሎ ሊያስረዳት ይችላል ፤ ልጇሽን ማጣት ስለለብሽ ነው፤ እሷንም ይቅርታ የሚጠይቅበት አጋጣሚ አለ ፤ አጋጣሚ ደግሞ እንደዚህ ሆኖ አዉትካሙ ባያምር መጀመሪያም የታገልኩት ላንችነው ይህ እንዳይከሰት ነዉ፤

ጠያቂ: ሳናነሳው የቀረ ነገር ግን ለጥናቱ ይጠቅማል የምትለው/ይዉ ሀሳብ ካለ የመጨረሻ እድል ልስጥህ/ሽ?

ተሳታፊ: እንደዚህ አይነት ነገር ሊመጣ የሚችለዉ ከባለሙያዉ ከድካም አንጻር ፤ ካለዉ የኬዝ ፍሎዉ አንጻር፤ የባለሙያዉና የእናቶች ብሮፖርሽን ተምጣጣኝ ስላልሆነ እንጂ የሚመጣ ማንኛዉም እናት ተገቢዉን አገልግሎት አግኝቶ መሄድ እንዳለበት ማንኛዉም ሰው ያምናል።

Type of data: Qual

የቃለ መጠይቁ ቀን:24/04/2016

የተሳታፊ: KII 3

የቃለ መጠይቁ ቆይታ:08:35 ሰከንድ

ጠያቂ:አክብሮት የተሞላበት የእናቶች እንክብካቤ ለእርስዎ ምን ማለት እንደሆነ ሀሳብዎን ያጋሩን? በእርስዎ አስተያየት፣ በዚህ የጤና ተቋም ውስጥ የሚሰጠውን አክብሮት የተሞላበት አገልግሎት ጥራት እንዴት ያዩታል? ከመልስዎ ጀርባ ያለውን ምክንያት ቢያብራሩልኝ?

ተሳታፊ: የ እናቶች ሲ አር ሲ የተሞላበት አገልግሎት አሰጣት ሙሉበሙሉ በሚባል ደረጃ አደለም፤

Interviewer: አክብሮት የተሞላበት የእናቶች እንክብካቤ እንደሰጠህ/ሽ እራስህን/ሽ አግኝተህ/ሽ ታውቃለህ/ሽ? ከሆነስ መቼ እና እንዴት ?

ተሳታፊ: ይህ የሆነበት ደግሞ ኬዝ ሲበዛብን አንድ ባለሙያ ሶስት አራት አልጋ ሲይዝ በዛ ሰዓት ሁሉንም እናቶች እነሱ በፈለጉ ሰዓት ላናያቸው እንችላለን፤ ባልፈለጉበት ታይም ብናያቸው ፍቃደኛ አይሆኑም፤ እኛን እንደመጥፎ አስገድደን እንደምንሰራ ሊያዩን ይችላሉ፤ ሌላው ማታ ላይ እንቅልፍ አለ ድካም አለ በዛ ሰዓት ሲ አር ሲ የተሞላበት አገልግሎት ላንሰጥ እንችላለን ሌላው ሆስፒታሉ ላይ ያለዉ ባለሙያ እና ኬዙ ማይገናኝ ነው ፤ በዛ ሰዓት ስራህን ነው እንጂ ሲአርሲ ላትሰጥ ትችላለህ፤ አንዳንዴ ደግሞ ሃላፊህ ጋር አለመስማማት፤ ጥቅም አላስከብርልህ ሲል፤ የሆነነገር ማግኘት ፈልገህ አላገኝ ስትል የዛን ቁጭት እናቶች ጋ ሲ አር ሲውን ላንተገብር እንችላለን ብዙጊዜ በኔ ማየዉ ፤ አንዳንዴ ደግሞ የ ቤተሰብ ችግር ሲኖር ሳትረጋጋ፤ ፈቃድ ሳይኖርህ ግባ ስለምትባል ብቻ የቤትህን ጸባይ እናቶችጋ ልታሳይ ትችላለህ ።አንዳንዴ እናቶችን ክብር የማንጠብቅበት ኤክስተርናል የሆነ ነገር ስለሚኖር እንጂ ፤የእነሱን ክብር መጠበቅ አልችል ብለን አደለም፤ እዚህ ሆስቢታል እንደምታዉቀዉ የባለሙያ እጥረት አለ፤ የማቴሪያል አቅርቦት እጥረት አለ፤ በዛ በዛ ብዙ ታማሚዎች ይንገላታሉ፤ የባለሙያ እጥረት አለ ማለት አንድ ባለሙያ ሶስት አራት አልጋ ይዞ ወይ ሶስት አራት ፔሸንት ይዞ የእዛን ሁሉ ጸባይ መቻል ከባድ ነው፤ በዛላይ ድካም አለ ፤ ድካሙ በጨመረ ቁጥር የእነሱን ክብር መጠበቅ አትችልም ይከብድሃል፤ ከግባት አንጻር ስናየዉ ደግሞ በምትፈልገዉ ጊዜ የምትፈልገዉን ማቴሪያል ካላገኘህ ለማስተናገድም ዎደኋላ የምትልበት ነገር ሊኖር ይችላል፤ ማድረግ ያለቢህን ነገር ላታደርግ ትችላለህ፤ በመሃል ምትናገረው ነገር ላይስማማት ይችላልብየነው ማስበው፤ ሌላው ስራሲበዛ

ድካም ሲኖር ምትናገረዉን አታዉቅም፤ የእነሱን ፍላጎት አንጠብቅም፤ ስራችንን ብቻ ምናይበት ሁኔታ ይኖራል ፤

ጠያቂ: በአንተ አስተሳሰብ አንዲት እናት በምትወልድበት ሰዓት በአክብሮት አገልግሎቱን እንድታገኝ/እንዳታገኝ ሊያደርጉ የሚችሉ ተያያዥ ጉዳዮች ምን ምን ሊሆኑ ይችላሉ ብለህ ታምናለህ? እንዴት?

ተሳታፊ: እንደዚሁ በጽንሱ ላይ መታፈን ሲኖር ወይም እሷ ኮምብሊኬሽን ሲያጋጥም የእሷን ፍላጎት ላንጠብቅ እንችላለን ፤

ጠያቂ:የወሊድ አገልግሎቱን የሚሰጠው/ባለሙያው አንዲት እናት በምትወልድበት ሰዓት ስነ-ምግባር በጎደለው ሰብዕና ሲያስተናግድ ተይዞ ተጠያቂ የሚሆነው እንዴት ነው?

ተሳታፊ: እንደ እኔ ቅጣትን አስቤ አደለም የምሰራው፤ በቃ መስራት ስላለብኝ ነው፤ ተጠያቂነት ሚኖረው ወይ ከልጇ ወይ ከናትይዋ ችግር ሲኖረው ፤እኛም እኮ ጸባያችን ጥሩ ማይሆነው ለእሷ ብለን ነው፤ ችግር እንዳይፈጠር ብለን እንጂ ተጠያቂነትን አስበን አደለም ፤

ጠያቂ: ሳናነሳው የቀረ ነገር ግን ለጥናቱ ይጠቅማል የምትለው/ይዉ ሀሳብ ካለ የመጨረሻ እድል ልስጥህ/ሽ?

ተሳታፊ: ሌላው የሚድዋይፍ ባለሙያ ታዉቀዋለህ የስራ ሎዱ ፤አክብሮቱ፤ ሪስኩ ስታየዉ ከባድ ነው፤ የትምህርት እድል የለም ፤ ማደግ ባለብህ ሰዓት አታድግም፤ ኑሮህን ለማመቻቸ ስትል የሆነበታ ብቻ መስራት የለብህም ፤መቀያየር አለብህ ፤ባለሙያዉም በዛልክ ሞራሉ እየቀነሰ እየቀነሰ ነው የሚሄደዉ፤ እነዚህ ነገሮች ቢሟሉ ባለሙያዉ የፈለገዉ ቢደረግ ይሄ ነገር ጥሩ ይሆናል ብየ አስባለሁ ፤ማቴሪያል ቢሟላ ፤ባለሙያ ቢጨመር፤ ያንተን ፍላጎት ቢያሟሉለህ በቻሉት መጠን፤ ጥሩ ይሆናል ብየ አምናለሁ።

Type of data: Qual

የቃለ መጠይቁ ቀን:24/04/2016

የተሳታፊ: KII 4

የቃለ መጠይቁ ቆይታ:12:10ሰከንድ

ጠያቂ:አክብሮት የተሞላበት የእናቶች እንክብካቤ ለእርስዎ ምን ማለት እንደሆነ ሀሳብዎን ያጋሩን? በእርስዎ አስተያየት፣ በዚህ የጤና ተቋም ውስጥ የሚሰጠውን አክብሮት የተሞላበት አገልግሎት ጥራት እንዴት ያዩታል? ከመልስዎ ጀርባ ያለውን ምክንያት ቢያብራሩልኝ

ተሳታፊ: በኛ ተቋም አክብሮት የተሞላበት የእናቶች ጤና አገልግሎት አሰጣት በመካከለኛ መጠን ነው የማየዉ ፤አክብሮትየተሞላበት የእናቶች ጤና አገልግሎት አሰጣት ከሰዉሰው ይለያያል፤ ግን በቂ የሆነ አክብሮት የተሞላበት የእናቶች ጤና አገልግሎት ሰተናል የሚል የለኝም፤

ጠያቂ: አክብሮት የተሞላበት የእናቶች እንክብካቤ እንደሰጠህ/ሽ እራስህን/ሽ አግኝተህ/ሽ ታውቃለህ/ሽ? ከሆነስ መቼ እና እንዴት ?

ተሳታፊ: ከስራ ብዛት አንጻር እኛ አካባቢ፤ እኛ አካባቢ የሚወልዱ እናቶች ያልተማሩ እናቶች ይበዛሉ መግባባት እንቸገራለን፤ አስታማሚዎቹ ጋር መግባባት እንቸገራለን፤ እናቶች የሚፈልጉት እንደመጡ መዉለድ ሊሆን ይችላል፤ እነሱን ብቻ እንድናያቸዉ ሊሆን ይችላል፤ እነሱ ሚፈልጉትን ብቻ እንዲሆንላቸዉ ስለሚፈልጉ አለመግባባት ይፈጠራ፤ እኛ ጋ የስራ ጫና አለ ከዛ አንጻር እናቶችን ርስቤክት ላናደርግ እንችላለን እንደዛ ብየነው ማስበው ፤ አክብሮት የተሞላበት የእናቶችጤና አገልግሎት ሰጥቻለሁ ብየማስበው በደንብ አክብሮት ሰጥቸ መብታቸዉን አክብሬ የእነሱን ፍላጎት ሳልጋፋ ወጣ ያለ ንግግር ሳንነጋገር ሳላመነጫጨቅ በሰላም ተግባብተን ማድረግ ያለብኝን ስራሰርቸ እስከመጨረሻዉ ድረስ ትክክለኛዉን ነገር እያደረግሁ ከጨረስሁ ነው አድርጌአለሁ ብየ የማስበው፤ ከዛዉጭ በመሃል ሰጣገባ አለመግባባት የተፈጠሩባቸዉን ሁኔታዎቸ አገልግሎቱን አልሰጠሁም ብየማስብባቸው ሁኔታዎች ናቸዉ፤ ለምሳሌ እናቶች ለእውለድ እኛጋ ይመጣሉ የተወሰነ ሰዓት እቤታቸዉ እቆዩ በኋላ ይህ እኛ የዉሽት ምጥ የምንለዉ ሰዓት ነው፤ የተዎሰነ ሰዓት አይተዉ ሰዓቴ ደርሷል ብለው ዎደኛ ከመጡ በኋላ ልክ እንደደረሱ መዉለድ ይፈልጋሉ፤ እቤታቸዉ ያለዉን ሰዓት ይቆጥሩታል ፤ ልክ እንደደረሱ ምጥ እረዘመብኝ ብለው ነው ሚያሰቡት፤ላስረዳት ስሞክር አንግባባም ፤ በዛ አለመግባብት ይፈጠራል፤በዛ የመሰላቸት እና ባህሪየ የመቀየር ነገር ይኖር፤ ከዚህ በኋላ ያልኩሽን

ነገር አድርጊ ማለት ይኖራል፤ሌላው እስታፍ ጋር አለመግባባት ካለ እና ተቋሙ ሰራተኞችን ፕሮሞት የማያደርግ ከሆነ ፤ የትምህርት እድል ከሌለ፤ ጥቅማጥቅም ከሌለውሰራው በባህሪዉ አድካሚ እና አሰልቺ ነው፤ ባለሙያው ሰራውን የመጥላት እና የመሰልቸት ሁኔት ይኖራል ፤ሌላው ስራው ማታላይ ይበዛል ሚያድረው ሰው ግን ትንሽ ቁጥር ነው በዛ ጊዜ አንድ ሰው ሶስት አራት ኬዝ በተመሳሳይ ጊዜ ሊይዝ ይችላል፤ በዛ ጊዜ አክብሮት የተሞላበት አገልግሎት ላንሰጥ እንችላለን፤ ሌላው በቂ ግባት የለም ብዙ ማቴሪያሎችን ዉጭነው የምናስገዛው፤ የሚመጡ ወላዶች ደግሞ አገልግሎቱ ነጻ እንደሆነ ነው የሚያውቁት ሲመጡ መግዛት ይቸገራሉ፤ ብሮሲጀር ለመስራት ፈልገህ እቃ ከውጭገዝተው ብር ፈልገው ነው የሚያመጡልህ፤ በማቴሪያል ችግር ምክኒያት መስራት እየፈለግህ ፤የፈለግህዉን ስራ እንዳትሰራ ይገድብሃል ይሄ ትልቅ ችግር ነው፤ ከአለቆች ክትትል ማነስ እስታፍ ወጣ ገባ ማለት ነገር አለ በጣም እንግዲህ ፈሪ ሰው ረጂም ሰዓቱን እዚህ ያሳልፋል ግዴለሽሰዉደግሞ ይወጣል እና የዛን ሰው ሁሉ ዎላድ እየመጣ ዝም ስለማልል ትሸፍናለህ ስትሸፍን ስራውን ጥላቻ ይኖርሃል እ ስራዉም ይደብርሃልበዛላይ አንተ እንደዛ እየሰራህ በሚጠፋውሰውላይ ምንማይነት እርምጃተደርጎ አላየነም እና እና እንደዛ ሲሆን አንተ ግዴለሽ የመሆን ነገርና ስራውን የመጥላት አይነት ነገር ብሰራም ባልሰራም ለውጥየለዉም አይነት ነገር

ጠያቂ: በአንተ አስተሳሰብ አንዲት እናት በምትወልድበት ሰዓት በአክብሮት አገልግሎቱን እንድታገኝ/እንዳታገኝ ሊያደርጉ የሚችሉ ተያያዥ ጉዳዮች ምን ምን ሊሆኑ ይችላሉ ብለህ ታምናለህ? እንዴት?

ተሳታፊ: ጽንሱ የመታፈን ነገር ሲኖረው የጽንሱ የልብ ምት ተልተሎ መስማት ይኖርብኛል፤ በዛ ሰዓት ህመም ላይ ስለምትሆን የጽንሱንን የልብ ምት እምቆጥርበት ጊዜ ላይመቻት ይችላል፤ በዛ ሰዓት አስገግጀ ወደምፈልገው ፖዚሽን አደርጋት ላይመቻት ይችላል ፤ ይሄን የማደርገው የጽንስ መታፈኑ የተበባሰ ደረጃ ሳይደርስ ልጇን ማትረፍ እማትረፍ ነው በዚህ ሁኔታ ላትረዳኝ ትችላለች በዚህም አለመግባባት ይፈጠራል፤ ብዙ ጊዜ በጥሩ ሁኔታ አላስተናገዱኝም ብለው የሚያስቡ እናቶች በመጀመሪያ ደረጃ ምጥ የመጡ እናቶች ናቸው፤ በመጀመሪያ ደረጃ ምጥ የመጡ እናቶች አልጋ ሰጥተን ከመረመርን በኋላ ኦብስታትሪክ የሆነ ችግር ከሌላቸው በቀር በየ አንድ ሰዓቱ ነው ምርመራ የምናደርገው፤ አብረው የተኙ እናቶችን በየ ስላሳ ደቂቃው እና በየ አስራምስት ደቂቃው በምንከታተልበት ጊዜ ካለመረዳት የተተሁ አይነት ስሜት ሊኖራት ይችላል። ስለዚህ በመጀመሪያ ደረጃ ምጥ የመጡ እናቶች ብዙዉን ጊዜ የተተሁ አይነት ስሜት ይኖራቸዋል ፤

ጠያቂ: የወሊድ አገልግሎት የሚሰጠውን ባለሙያ የስራ የቅርብ አለቃው ባያከብረው በወላድ እናቷ ላይ ምን ያስከትላል ብለህ ታስባለህ? እንዴት?

ተሳታፊ: እኔ በቅርብ አለቃየ ባልከበር ለኔ ኪብር ባይኖረው ታችላይ ወርጀ እኔ ደግሞ ከኔ በታች ያሉትን ወይም ደግሞ አገልግሎት የምሰጣቸዉ ሰዎችላይ ክብር መስጠት አልችልም እኔ ተጎድቻለሁ መጀመሪያ ተበሳጭቸአለሁ እሱ በሚያሳየኝ ክብርና ለስራየም አገልግሎት ለምሰጣቸዉ ሰዎች ክብር መስጠት እችገራለሁ በቅርብ አለቃየ እንደዚህ አዪነት ነገር ከደረሰብኝ

ጠያቂ: የወሊድ አገልግሎቱን የሚሰጠው/ባለሙያው አንዷት እናት በምትወልድበት ሰዓት ስነ-ምግባር በጎደለው ሰብዕና ሲያስተናግድ ተይዞ ተጠያቂ የሚሆነው እንዴት ነው?

ተሳታፊ: እኔ በማዉከዉ ሁኔታ ተጠያቂነትየለም ተጠያቂነት ቢኖር ጥሩ ነው እኛ በባህሪአችን ምንጠየቅበት ሁኔታ ሲኖር ነው ትኩረት ሰጠን የምንሰራው እና ትጠያቂነት ቢኖር እና ከጀርባዉ ያለዉ ያሰው ቢጠና ያን ስራ እንዳይሰራ ያደረገው እስካሁን ያዎራናቸዉን ነገሮች ማስተካከል ቢቻል እነዚህ ነገሮች ቢስተካከሉ የጤና ባለሙያዉ ማያስተካከል ከሆነ ተጠያቂነት ቢኖረው የእናቶችን አገልግሎት የተሻለ ማድረግ እንችላለን ብየ አስባለሁ

ጠያቂ: ሳናነሳው የቀረ ነገር ግን ለጥናቱ ይጠቅማል የምትለው/ይዉ ሀሳብ ካለ የመጨረሻ እድል ልስጥህ/ሽ?

ተሳታፊ: መጨመር የምፈልገውለእናቶችክብር የተሞላው አገልግሎት እንዳንሰጥ ከሚያደርጉ ነገሮች ቅድም ያልጠቅስሁልህ ለወሊድ የመጡ እናቶች አንተ ወይም እኔ ጤና ባለሙያዉ ይዟቸዉ ሚፈልጉት ሌላሰው ማምጣት ነው በሶሻል አቴዳንቶቻቸዉ ይረብሹሃል እዛ ሚሰራ ሰው ሶሻል ጣልቃማስገባት ነገር ይኖራል እዛላይ አለመግባባት ነገር ይፈጠራል እሱም ለእናቶች ክብር ያለው አገልግሎት እንዳንሰጥ አንዱ ምክኒያት ነው ብየአሚናለሁ አንተ በራሰህ በተማርከው በምታውቀው እየሰራህ እየሰራህ በመሃል እንዲ ብታደርግ እንዲ ቢታደርግ እንዲ ባታደርግ ሲልህ እናቶችላይ ያልተገባ ነገር እንድናደርግ ያደርጋል ብየ አስባለሁ እንደዚህ አዪነት ነገር ጣልቃሚገባበት ነገር ቢቀሩ የስራ ጫና ለመቀነስ ሚጠፉ ባለሙያዎችላይ ቁጥጥር ቢደረግ ለዉጥ ይመጣል ብየ አሚናለሁ

Type of data: Qual

የቃለ መጠይቁ ቀን:25/04/2016

የተሳታፊ: KII 5

የቃለ መጠይቁ ቆይታ:06:14 ሰከንድ

ጠያቂ: አክብሮት የተሞላበት የእናቶች እንክብካቤ ለእርስዎ ምን ማለት እንደሆነ ሀሳብዎን ያጋሩን? በእርስዎ አስተያየት፣ በዚህ የጤና ተቋም ውስጥ የሚሰጠውን አክብሮት የተሞላበት አገልግሎት ጥራት እንዴት ያዩታል? ከመልስዎ ጀርባ ያለውን ምክንያት ቢያብራሩልኝ?

ተሳታፊ: የ እናቶች ጤና አገልግሎት በሚባለው ልክ አንሰጥም

ጠያቂ: አክብሮት የተሞላበት የእናቶች እንክብካቤ እንደሰጠህ/ሽ እራስህን/ሽ አግኝተህ/ሽ ታውቃለህ/ሽ? ከሆነስ መቼ እና እንዴት ?

ተሳታፊ: ምክኒያቱም ኬዝ ይበዛል ባለሙያው እና ሚመጣዉ ኬዝ ፍሎው እኩል ስላልሆነ ተገቢዉን አገልግሎት ላያገኙ ይችላሉ ነገር ግን በሚባለው ልክ ማገልገል የማይቻልበት ሚክኒያት የሰው ብዛት የባለሙያውና ሬሾ ስለሚለያይ አገልግሎቱ ቢሰጥም ብቁ አደለም፤አንዳንዴ እኔ በዚህ ሆስፒታል ሳገለግል ብዙ ጊዜየ ነው የትምህርት እድል አላገኘሁም አራት አመት ሁኖኛል ምሰራው እና ሚከፈለኝ ልዩ ነት አለዉ ወይ አልተማርኩ ያና ይህ ሲደመር አንዳንዴ ትበሳጨለህ ማናምን በዛልክ ነው ከተቋሙ አንጻር ትሬኒንጎች አይሰጡም አብዴት የሆኑ ነገሮች አይደርሱህም አድሬስ አይደረጉም ተምረን እንደዎጣን በዛኛው ነው ምትሰራ እንጂ በየጊዜው አብዴት የሆኑ ነገሮች በትሬኒግ መልክ አይሰጡም አንዳንድ ግባቶችሊያጥሩ ይችላሉ ግባት ስንል አንዳንድ ጊዜ ለምሳሌ እስክሪን ላታገኝ ትችላለህ አንድ ታካሚ ለማየት ሌበር ላይ ሁነህ እስክሪን ሳታደርግ ነው ምታየዉ ፌታል ህርትቢት ስታዳምጥ እስክሪን አድርገህ ነው ግን እስክሪን የለም እ ኮችላይ ስታዋልድ ሁለት ሶስት ኮች ካለ እስክሪን አድርገህ ነው፤

ጠያቂ: በአንተ አስተሳሰብ አንዲት እናት በምትወልድበት ሰዓት በአክብሮት አገልግሎቱን እንድታገኝ/እንዳታገኝ ሊያደርጉ የሚችሉ ተያያዥ ጉዳዮች ምን ምን ሊሆኑ ይችላሉ ብለህ ታምናለህ? እንዴት?

ተሳታፊ: ሚክኒያቱም ደግሞ አለመግባባት ይኖራል በሽተኛዋ ሚጠቅማተን ነግረሃት አይ መብቴ ነው ብትል መብቷን ልትጋፋ ትችላለህ እየየህ ይሄን ሪስክ መዉሰድ ስለለለብህ እሷም በትንሽደቂቃ ያንን ነገር ልታጣው ስለምትችል ለምሳሌ አንድ እናት ልጇ እየታፈነ መብቷ ስለሆነ በፈለገችዉ መተኛት በግራ ጎን መተኛት እያለባት እኔ መብቴ ስለሆነ በቀኝ ጎኔ ነው የምተኛው ብትል አንዳንዴ ትጋፋታለህ ወይ ደም እየፈሰሳት ኦብረሽን ይሰራልሽ በጣም ደም እየፈሰሰሽ ነው ተብላ አይ አልሰራም ብትል አንዳንዴ እሷን ለማትረፍ መብቷን ልትጋፋ ትችላለህ

ጠያቂ:የወሊድ አገልግሎቱን የሚሰጠው/ባለሙያው አንዲት እናት በምትወልድበት ሰዓት ስነ-ምግባር በጎደለው ሰብዕና ሲያስተናግድ ተይዞ ተጠያቂ የሚሆነው እንዴት ነው?

ተሳታፊ: ተጠያቂነት ሲኖር ስራ ይሰራል ደግሞ ትጠያቂነት ባለመኖሩ ስራ አይሰራም ማለት አይቻልም እሱ እንዳለ ሆኖ ሆስፒታሉ ወይም ያተቋም ሚሰጠው አገልግሎት ባለሙያውን ያማከለ መሆን አለበት ለመጠየቅ መጠየቂያዉን መስፈርት አሟልቶ ልክ አድርጎ ሲሰጥነው አንድ ሰው ሶስት አራት እናት እያዋለደ በዛልክ ትጠየካለህ ወይም ወ ይም ስምንት ዘጠኝ ኬዝ ይዞ አንድ ሰው በዛልክ ተጠያቂነት ስታበዛበት ትንሽ ይደብራል ግ ን አንድላንድ ሬሾ አድርገን ኖሮ ላልሰራው ነገር ትጠያቂ ብታደርገው መልካም ነው ብየ አስባለሁ ግ ን ሚሰራው ስራ እና በዛልክ በዛልክ ተጠያቂነቱን ብታደርገው ሰዎች ሚያገኙትን አገልግሎት በጣም ይቀንሰዋል ብየ አምናለሁ

ጠያቂ:ሳናነሳው የቀረ ነገር ግን ለጥናቱ ይጠቅማል የምትለው/ይዉ ሀሳብ ካለ የመጨረሻ እድል ልስጥህ/ሽ?

ተሳታፊ: ቢጨመር ብየ የማስበው ባለሙያው በደንብ ይሄንን ነገር ቀጥታ ቢወስድእና ቢሰራ ፔሸንቶቹ እና ማህበረሰቡ ይህ አዌርነስ ቢፈጠር ፤ ለምሳሌ ቅድም እንዳልኩህ በቀኝ ጎኔ መተኛት መብቴ ነው ስትል ግዴታም እንዳለ እንዲህ አይነት ነገር ሲሆን አድዳንዴ ባለሙያ የራሱን እርምጃ ይዎስዳልሚለው ነገር ፔሸንቶች ቢያውቁ ወይም ታካሚዎች አገልግሎቱን ሲያገኙ እዛ ጤና ተቋም ዉስጥባለሙያው የራሱን እርምጃ ይዎስዳል ሚለዉን አብሮ ተካትቶ እነሱ ቢያዉቁ መብት እንዳለ ሁሉ ግዴታም እንዳለ ጥሩ ነው ብየ አስባለሁ

Type of data: Qual

የቃለ መጠይቁ ቀን:25/04/2016

የተሳታፊ: KII 6

የቃለ መጠይቁ ቆይታ:05:50 ሰከንድ

ጠያቂ:አክብሮት የተሞላበት የእናቶች እንክብካቤ ለእርስዎ ምን ማለት እንደሆነ ሀሳብዎን ያጋሩን? በእርስዎ አስተያየት፣ በዚህ የጤና ተቋም ውስጥ የሚሰጠውን አክብሮት የተሞላበት አገልግሎት ጥራት እንዴት ያዩታል? ከመልስዎ ጀርባ ያለውን ምክንያት ቢያብራሩልኝ?

ተሳታፊ: ያው እንግዲህ የእናቶች አክብሮት ሰጥቶ ስራን መስራት በአግባቡ ከበፊት ጀምሮ አለ በመጠኑም ቢሆን ሙሉ መቶ ፐርሰንት አክብሮት የተሞላው ነው ለማለት ባይቻልም አክብሮት የተሞላው ስራ ሁሌም እንደሚሰራ ነው የምናዉቀው እኛ የምንሰራዉም

ጠያቂ: አክብሮት የተሞላበት የእናቶች እንክብካቤ እንደሰጠህ/ሽ እራስህን/ሽ አግኝተህ/ሽ ታውቃለህ/ሽ? ከሆነስ መቼ እና እንዴት ?

ተሳታፊ: ብዙ ምክኒያቶች ሊነሱ ይችላሉ እኔ ግ ን የተዎሰኑትን ነው ምጠቅስለህ ለምሳሌ የስራ መብዛት ጋር የስራ ሎድ አንዱ ሊሆን ይችላል እ ታይም ለምሳኤ ኦበር ናይት እና ደይ ታይም ያለው እሱሊሆን ይችላል እ ምት ሰራበት ጤና ተቋም ኢንፍራስትራቸር አለመመቻቸት እንደምታውቀው ከክፍያ አንጻርም ሰው እንደዚህ እንትትን ማለንት እነሱ እነሱ በዋነኝነት የሚነሱ ነገሮች ናቸዉ

ጠያቂ: በአንተ አስተሳሰብ አንዲት እናት በምትወልድበት ሰዓት በአክብሮት አገልግሎቱን እንድታገኝ/እንዳታገኝ ሊያደርጉ የሚችሉ ተያያዥ ጉዳዮች ምን ምን ሊሆኑ ይችላሉ ብለህ ታምናለህ? እንዴት?

ተሳታፊ: አንዳንድ ደግሞ በስራህ ሁኔታ ለልጅ እና ለናት ባለው እንዳልኩህ ዉቴት ለማግኘት ትሩዉቴት ስንልየእሷን እንትትን ማንጠብከው ሊኖር ይችላል ለምሳሌ በወሊድ ሰዓት የልጅ መታፈን ሊኖር ይችላል እንደዚህ እንደዚህ አይነት ነገርች ሲኖሩ ምጡ ደርሶ ለመዉለድ በቀረበች ሰዓት ልጁ መታፈን ሂደት ላይ እያለ መችም እሷን ርስቤክት ለማድረግ ምንቸገርበት ሁኔታዎች አሉ እና እነሱ እነሱ አይነት ነገሮች ሙሉ

እንዳይሆን የሚያደርጉ ነገሮች መስለው ይታያሉ በንደዚህ አይነት ነገሮች አሁን ለምሳሌ እንዳልኩህ በልጅ መታፈን እየደማች ሊሆን ይችላል እንደዚህ እንደዚህ አዪነት የስራ እንትኖች እንዳት ሰጥ ያደርጉሃል እንግዲህ እኛም ጋ ያለው ሁሉም ነገር መቸም እየደማች ሁሉን ነገር ለማድረግ ቢናሰብ ሰትዮይቱን በሂዎት ልናገኛት አንችልም ስለዚህ አንዳንድ እንደዚህ አይነት ነገሮች ሲያጋጥሙ ክብር ምናምን ሚለው ነገር አቢሮ ላይሀድ ይችላል

ጠያቂ:የወሊድ አገልግሎት የሚሰጠውን ባለሙያ የስራ የቅርብ አለቃው ባያከብረው በወላድ እናቷ ላይ ምን ያስከትላል ብለህ ታስባለህ? እንዴት?

ተሳታፊ: አዎ ሚናልባት እኔ ገጥሞኝ አያዉቅም ሊኖር ይችላል ብየ አስባለሁ ምክኒያቱም አንዳንድ መነጋገር ሲኖር ብስጭት ይኖራል ዉስጥህ ጥሩ ላይሆን ይችላል ውስጥህ ጥሩ ሳይሆን ኦፒዲ ላይ ቁጭ ብትል እዛሰው ጋር ያለህ ኝኙነት ጥሩ ይሆናል ቢየ አላስበም መጀመሪያ አምሮህ የተረጋጋ ሆኖ ምት ሰራው እና ተነጋግሮ ዎይም ከጸብ ተመልሰህ ምትሰራው ስራ እኩል ነው ብየ አላስብም እንደኔ ተጽኖ አለው ብየ ነው ማስበው

ጠያቂ:የወሊድ አገልግሎቱን የሚሰጠው/ባለሙያው አንዲት እናት በምትወልድበት ሰዓት ስነ-ምግባር በጎደለው ሰብዕና ሲያስተናግድ ተይዞ ተጠያቂ የሚሆነው እንዴት ነው?

ተሳታፊ: እኔ ተጠያቂነት አለመኖሩ አንድ ፋክተር ነው ብየ አስባለሁ ተጠያቂነት መኖር አለበት ብየ አስባለሁ ምክኒያቱም አንድ ሰው እርስቤክትፉል ወይም ክብር ከሌለው ሳይጠየቅ ቢቀር ሌላ ያነን እየቸመረው ይሄዳል ምናገባኝነት ወይም ማንም አይጠይቀኝም አይነት ነገር ይኖራል ብየ አስባለሁ ነገር ግ ን ኮሜንት ቢደረግ እና እንዲስተካከል ቢነገረው ተሳስቻለሁ ስተት ነበር እራሱን ሚዎቅስበት ወይ ይኖራል ከዛም እራሱን ያስተካክላል ዎደፊት ሌሎች እናቶች ጋር ያለዉን ያስተካክላል ብየ አስባለሁ

ጠያቂ: ሳናነሳው የቀረ ነገር ግን ለጥናቱ ይጠቅማል የምትለው/ይዉ ሀሳብ ካለ የመጨረሻ እድል ልስጥህ/ሽ?

ተሳታፊ: እንደእኔ ይህ አር ኤም ሲ በደንብ ለጤና ባለሙያዎች ተሰጥቶ በትሬኒግ አይነት ነገር መጥቶ ለሁሉም ባለሙያ ተደርስሽ ቢሆን መጻፍትም ሞጁሎችም ተዘጋጅተዉ በየክላሱ ቢቀመጡ እና እንደሌላው ኳሊቲ እንደምንለው እንዳንድ ተካትቶ ያለው ነገር ተደርሽ ቢሆን ያለው ነገር ተደርሽቢሆን ጥሩ ነው ብየ አስባለሁ

Type of data: Qual

የቃለ መጠይቁ ቀን:**25/04/2016**

የተሳታፊ: **KII 7**

የቃለ መጠይቁ ቆይታ:**11:08 ሰከንድ**

ጠያቂ: አክብሮት የተሞላበት የእናቶች እንክብካቤ ለእርስዎ ምን ማለት እንደሆነ ሀሳብዎን ያጋሩን? በእርስዎ አስተያየት፣ በዚህ የጤና ተቋም ውስጥ የሚሰጠውን አክብሮት የተሞላበት አገልግሎት ጥራት እንዴት ያዩታል? ከመልስዎ ጀርባ ያለውን ምክንያት ቢያብራሩልኝ?

ተሳታፊ: እንግዲህ ከጤና ተቋም ዉስጥ ከሚሰጡ አገልግሎቶች እስቤሻሊ ለእናቶች አካባቢ ሚሰጡ አገልግሎትች ከአክብሮት አንጻር ምንድን ነው ያለው ነገር በሆስፒታላችን አንደኛ ኦልሞስት በሚባል ደረጃ ሁሉም ባለሙያ አክብሮት አለው ለእናቶች የእናቶችን ክብር በጠበከ መልኩ አገልግሎቶችን እየሰጠ ይገኛል ብለን እናስባለን።

ጠያቂ: አክብሮት የተሞላበት የእናቶች እንክብካቤ እንደሰጠህ/ሽ እራስህን/ሽ አግኝተህ/ሽ ታውቃለህ/ሽ? ከሆነስ መቼ እና እንዴት ?

ተሳታፊ: አድዳንዴ ምንድን ነው ከአክብሮት ጋር ተያይዘው ሚመጡ ነገሮች እንግዲህ አክብሮት ሲባል ብዙ ነገሮች አሉ ከኢንባይሮመንቱ አንጻር ከሆስፒታሉ ከአቅርቦት አንጻር ከጤና ጣቢያው ከጤና ተቋሙ ባለሙያዎች የድካም አንጻር እና የትሬኒግ እጥረትም ሊሆን ይችላል ለምሳሌ አንዳንዴ የስራ ጫና አለ እንደሚታዎቀው የስታፍ ቁጥር እና የታካሚዎች የእናቶች የእናቶች አለመመጣጠን ይኖራል በዚህ ግዜ ሊከሰቱ የሚችሉ ነገሮች ሚድን ነው ከስራ ብዛት አንጻር ሰው ነህ ልትደክም ትችላለህ እናቶችን የማመነጫጨቅ ከስራ ቢዚነት አንጻር ኬዝ ከሙብዛት ከመወጠር አንጻር ማመነጫጨቅ ሊኖር ይችላል አልፎ አልፎም የሚከሰት ነገር ነው ሌላው ምንድን ነው ከጤና ተቋሙ አክብሮት አንጻር ለምሳሌ በሚያዋልድበት ሰዓት በቂ የሆነ የኢንፌክሽን መከላከያ ነገሮች ላይኖሩ ይችላሉ ከቦት ከጫማ ከሚናሚን አንስቶ እነዛ ነገሮች ደግሞ በሚያጋጥምህ ኮንታሚኔሽን አንጻር ልትበሳጭ እና ኬዝ ማኔግ በምታድረርግበት ጊዜ ለኮንታሚኔሽን የማጋለጥቻንስ አለው እንደሚታዎቀው ማዋለጃ አካባቢ ያለ ነገር ነው ቢዙ ጊዜ ኮንታሚኔሽን አዴጠፋም እና የመነጫነጭ ክሊያንቶችን ያለማክበር ሊኖር ይችላል እንግዲህ በሆስፒታኡ ሆስፒታል ላይ ያለው ባለሙያ የትምህርት ደርጃችንም በዛው ልክ የተለያየ ሊሆን ይችላል ስለዚህ ካለን የእናቶች ዎይም ፔሽንቶቻችንን እንዴት ማኔጅ ማድረግ እንንደምንችል ያለማዎቅ እጥረት ሊኖር ይችላል

ወይም አካሄድ በራሱ አለማወቅ አንዳንዴ የእናቶችን ክብር ሊያደርግ ይችላል ስለዚህ በአጠቃላይ ምንድን ነው እንደየሰው የሚለያይ ሁኖ ነገር ግ ን ደግሞ ከሆስፒታል አገልግሎት አንጻር ጥራቱ ከሆስፒታሉ የተለያየ ነገር ሲመጣ በቂክብር ላይኖረው ይችላል ከዛዉጬኝ ያለው የሆስፒታሉ አገልግሎት አሰጣት አልሞስት ሁሉም እናቶች ባከበረ መልኩ ነው ቢየ አስባለሁ እንደኛ ሆስፒታል፤እኔ የማስታዉሰው የለኝም እኔ አልሞስት የሚመጡ እናቶችን በክብር ነው በአክብሮት አስተናግዳለሁ ቢየ ነው ማስበው ልክ እንደነገርኩህ አንዳንዴ በእናቶችላይ ሳይሆ ሆስፒታሉ ላይ ባለው ነገር የስራ ምቹነት አንጻር በስራ ምቹነት ካለመሆን አንጻር እራሴን ቢዚ የማድረግ እና እራሴን ምቹ ላያደርገኝ ይችላል ብየ የማስበው ሚንድን ነው በምታዋልድበት ጊዜ በቂ ያልሆነ እንዴው ከኢንፌክሽን ለመጠበቅ በሽታኮታሚኔሽን ከመሆን ጋር እነሱን ለመከላከል ከሚቀርብ ጊባት ጋ እጥረት አለ እና ብዙ ጊዜ በደም ስትረጭ ደም ሲነካህ እሱን ሳታደርግ ቀርተህ በዛ የተነሳ እራሰህን ለተለያየ በሽታ ስትጋልጥ ትንሽ አንተንም ከመጉዳት አንጻር በላይ እናቶችንም በደንብ ማኔጅ ያለማድረግ ነገር ይኖራል ስለዚህ የሆነነገር ሰርተህ ኮታ ስትሆን ግላብህን አዉልቀህ ልትዎጣ ትችላለህ ስለዚህ ከእንደዛ አንጻር ካልሆነ እኔ በራሴ በኩል ቅድም እንዳልኩህ ከምጸራበት እንባይሮመንት አንጻር ፤

ጠያቂ:በአንተ አስተሳሰብ አንዲት እናት በምትወልድበት ሰዓት በአክብሮት አገልግሎቱን እንድታገኝ/እንዳታገኝ ሊያደርጉ የሚችሉ ተያያዥ ጉዳዮች ምን ምን ሊሆኑ ይችላሉ ብለህ ታምናለህ? እንዴት?

ተሳታፊ: እንግዲህ ከናቲቱ አገልግሎት አንጻር ምልህ ሂሄ ነው ብየ ለምነገር ቢያቅጠኝም እንግዲህ እና የያዝነው በሽተኛ ነው ታካሚ ደግሞ ብዙ ፍላጎቶችን ብዙ ነገሮችን ይዞ ይመጣል ማለት ከጤና አንጻር የሚያስጨንቀው ነገር ይኖራል እርግዝናው ያስቸንቃታል ያረገዝኩት ልጂምን ይሆናል ቢለው ይሰጋሉ ምጡ በሰላም ያልቅልኛል አያልቅልኝም ሚለው ነገር ያስጨንቃቸዋል ስለዚህ እናቶች ጋም የመነጫነጨና ባለሙያን ያለማክበር ነገር ሊኖር ይችላል ከታካሚዎቻችን ነገር ግ ን እነሱን ነገሮች እንደባለሙያ ከበር አድርገን እስፔሻሊ ከምክር አገልግሎት እና አሰጣት አንጻር ከፔሽንት ተግባብቶ የመስራት ነገር ሊኖር ይችላል ስለዚህ ከ እናቶች ወይም ከታካሚዎች እንደዚህ የመነጫነጭ ነገር ይኖራል አንዳንዴ አገልግሎቱን እየሰጠሃት ኮብሬቲብ የማትሆን ከሆነ አሁን ባለሙያዎችን አንዲት እናት አገልግሎቱን ልሰጣት እያሰብህ አልረዳህ ስትል ብዙ መክረህ ቢዙ አድርገህ አልረዳህ ስትል እንደሰው የመናደድ እና የመነጫነጭ ነገር ክብሯን ያለመጠበቅ ሊኖር ይችላል ለምሳሌ የሆነ ሰርቢስ ርስጥሽ እያልካት እምቢ ስትልህ ለዛ ሰርቢስ የምት ሰጠው ኮምብሊኬሽን ያንሰርቢስ ባታገኚ የሚመጣዉን ኮምብሊኬሽን ታስባለህ ያን ሰርቢስ ደግሞ እሷ ለመቀበል

ፈካደኛ አልሆን ስትል እንደዛ አዪነት ነገሮች እናቶችን በምክር ባክብሮ ከማስረዳት ይልቅ ተነጫንጮ የመመለስ ነገር አንዳንዴ ይታያሉ

ጠያቂ: የወሊድ አገልግሎት የሚሰጠውን ባለሙያ የስራ የቅርብ አለቃው ባያከብረው በወላድ እናቷ ላይ ምን ያስከትላል ብለህ ታስባለህ? እንዴት?

ተሳታፊ: አስተዳድራዊ ጉዳይ የመጀመሪያዉ ነው አስተዳድራዊ ጉዳይ ስንል ደግሞ ከአለቃህ ጀምሮ በቅርብ ያለው የቲም ሊደርህ ይጀምራል ስለዚህ በአለቃህ ወይም በቲም ሊደርህ የሚደርስብህ አስተዳድራዊ ተጽኖ ኢንዳሬክትሊ ፔሸንቶቻችንም ይጎዳል ያማለት አንተ ፍሬ ስትሆን እና አምሮህ ንጹህ ሲሆን ነው ጥሩ ሰርቢስ የምትሰጥ ከዛዉጭ አንተ በቅርብ አለቃህ ማይረባ ተጽኖ ሲደርስብህ ከ አንተ ተጽኖ በላይ አልፎ እናቶችን የመገላመጥ እና በቂ የሆነ ያለመስጠት ይኖራል ይህ ምንም ጥርጥር የለዉም ይገጥማላ፤ አንተ በቅርብ አለቃህ በኩል ተጽኖ ደርሶብህ በዛ አለቃህ ዉስጥ ለሚመለከተው አካል እንኳን የሆነ ነገር አመልክተህ በቂ የሆነ አስተዳድራዊ ችግር ካልተፈታልህ ስራዉን እስከ መጥላት እና ዘገምተኛ እስከመሆን ሊደርስ ይችላል ያማለት ሚጠበቅብህን ነገር በትክክል ላጸራ ትችላለህ እናቶቻችንን በትክክል አክብሮት ላታደርግ ትችላለህ እና ከዛ አንጻር እኔ በበኩሌ አምሮየ ነጻ ሲሆን ነው ጥሩ አገልግሎት የምሰጠዉ እና በቅርብ አለቃ ተጽኖ ከደረሰብህ የምሰጠው አገልግሎት በዛልክ የቀነሰ ነው ሚሆንብኝ ብየ አስባለሁ፤

ጠያቂ: የወሊድ አገልግሎቱን የሚሰጠው/ባለሙያው አንዲት እናት በምትወልድበት ሰዓት ስነ-ምግባር በጎደለው ሰብዕና ሲያስተናግድ ተይዞ ተጠያቂ የሚሆነው እንዴት ነው?

ተሳታፊ: በበኩሌ ለአንድ ነገር ተጠያቂነት ካለ የመጀመሪያው የህግ የበላይነት ይቅድማል ስለዚህ በቅርብ አለካህ ለሚደርስብህ ነገር ካለ ስራህም አንተም አንደኛ ለደረሰብህ ነገር ለመተየቅ ወይም ለደረሰብህ ነገር ፍትህ መጠየቅ ትፈልጋለህ ያን ፍትህ ካገኘህ በስራህ ኮንጆ ምንም ሳይከፋህ እናቶችን ልታገለግል ትችላለህ ስለዚህ ከሚሰጥህ ፍትህ አንጻር ፍትህ ካለ በቂ አገልግሎት ይኖራል ብየ አስባለሁ

ጠያቂ: ሳናነሳው የቀረ ነገር ግን ለጥናቱ ይጠቅማል የምትለው/ይዉ ሀሳብ ካለ የመጨረሻ እድል ልስጥህ/ሽ?

ተሳታፊ: እንግዲህ የእናቶችን ክብር ከመጠበቅ አንጻር የማስበው ነገር ሚንዲን ነው ያው ባለሙያው ያው እንደነገርኩህ እናቶች ሲመጡ ህዞ ችግር ይዘው ነው ወደ አንተ የሚመጡት ስለዚህ እንደባለሙያ ደግሞ እነዛን ችግሮች ተረድቶ በቂ የሆነ ጥሩ አገልግሎት መስጠትን ይጨምራል።

ስለዚህ በተስሃለ መልኩ ይሄንን ክብር እንዳንሰጥ የሚያደርጉ ዘርፈቢዙ ችግር አለ እነሱ ችግሮች በግዜ ቢፈቱ ለምሳሌ አስተዳድራዊ ችግሮች የአቅርቦት እንግዲህ አቅርቦት ስንል ከትንሽነገር ይጀምራል እንግዲህ ያለንን እሪሶርስ እንኳን በትክክል መጥከም ሳንችል ቀርተን ስራ ክፍተት ይፈጠራል እና እንደዚህ አይነት ነገሮችን ከሪሶርስ አንጻር ሁሉ ከቅርብ አለቃህ ግር ሁነህ ከሚመለከተው አካል ጋር ተቀራርበህ እነዚህን ችግሮች ቢፈቱ የተሻለ የእናቶችን በአክብሮት የማስተናገድ እድሉ ሰፊ ነው ብየ አስባለሁ።

Type of data: Qual

የቃለ መጠይቁ ቀን:25/04/2016

የተሳታፊ: KII 8

የቃለ መጠይቁ ቆይታ:09:23 ሰከንድ

ጠያቂ: አክብሮት የተሞላበት የእናቶች እንክብካቤ ለእርስዎ ምን ማለት እንደሆነ ሀሳብዎን ያጋሩን? በእርስዎ አስተያየት፣ በዚህ የጤና ተቋም ውስጥ የሚሰጠውን አክብሮት የተሞላበት አገልግሎት ጥራት እንዴት ያዩታል? ከመልስዎ ጀርባ ያለውን ምክንያት ቢያብራሩልኝ?

ተሳታፊ: የእናቶች አክብሮት የተሞላው የጤና አገልግሎት በተመለከተ ያው ከሞላ ጎደል ጥሩ አገልግሎት እየሰጠን ነው ቢለን እናሰባለን ግ ን ደግሞ ሙሉ ለሙሉ ለማለት አስቸጋሪ ሁኔታዎች አሉ

ጠያቂ: አክብሮት የተሞላበት የእናቶች እንክብካቤ እንደሰጠህ/ሽ እራስህን/ሽ አግኝተህ/ሽ ታውቃለህ/ሽ? ከሆነስ መቼ እና እንዴት ?

ተሳታፊ: ቢዙ ነገሮች አሉ አንደኛ ነገር የእናቶች አገልግሎት እንደሚታዎቀዉ ነጻ ነው ግባቶች ለእናቶች የሚመጡት ቶሎ የማልቅ ሁሉም አይነት ግ ባቶች ያለመምጣት ነገር ስላለ በዚህ ሚክኒያት እናቶች ብር ይዘው ስለማይመጡ እሱን ግዙ ስንላቸዉ እንትን የማለት ለምሳሌ አንዷት ኦብረሽን የምትገባ እናት እስቲች ባለመኖሩ ምክኒያት ዉጭ ግዢ ስንላት በዛበዛ ዲሊያንስ አገልግሎቱን ሙሉ ለሙሉ ለምስጠት ሚያስቸግር አንዱ ምክኒያት የግባት ችግር ነው ሌላው ችግር ደግሞ የሙያተኛ እጥረት አለ ለምሳሌ እንደኛ ሆስፒታል በእናቶች ማዋለጃ ክፍል እኔ እንደምሰራበት ክፍል ሰባት አልጋዎች ምጣቸዉ ጀምሮአቸው መዉለድ እስከሚደርሱበት ግዜ የሚቆዩበት ሰባት አልጋዎች አሉ ግ ን ጠቅላላ ያለዉ የሙያተኛ ብዛት ሶስት ነው ያው በተማሪዎች እና በዩኒበርስቲ እስታፍ ነው የሚሸፈኑት እንግዲህ ሶስት ሙያተኛ ለሰባት አልጋሚለው እሱም አንድተግዳቶት ነው።ያው የእናቶች አገልግሎት አክብሮይ በተሞላበት መልኩ አድርጌ አለሁ ብየ የሚለው ቢዙ ጊዜ የእናትዮዋ እና የልጇ ሁኔታ እሱ ሰላም ከሆነ በዚኛዉ ጎኔ መተኛት እፈልጋለሁ መምበርከክ እፈልጋለሁ ያው ሌበር ፖዚሽንና ደሊበሪ ፖ ዚሽን የሚባሉ አሉ አንዷት እናት በፈለገችዉ ፖ ዚሽን ደሊበር ማድረግ ትችላለች በፈለገችዉ ደግሞ እስከ ሰከንድ እስቴጅ ሌበር ፖዚሽን ላይ መሆን ትችላለች እነሱን ፖዚሽኖች እንትን አእልም ቅድም እንዳልኩህ ምንም ነገር ሃብን ካልሆነ በልጁም በሷም እሱን ፖዚሽን እናስተቅማታለን ይህ መቆምጠት ምናምን ሚባሉ ነገሮች አሉ እሱን እንደርሴ ስለሆነ መናገር ምችለዉ እንግዲህ ከሰራሁ ሰባት አመት ሁኖኛል ቆንጥቸ አላዉቅም ምን አልባት ሰከንድ እስቴጅ ላይ ስትሆን ልጁ

እንዳይታፈን ፖዚሽን አልይዝ ስትል ፖዚሽን ያዥ ብሎ ከመናገር ዉጭ እንደዛ ብየ አላዉቅም እና ይሄን አገልግሎት ስንሰጥ ሌበር ፖዚሽን ወይም ደሊበሪ ፖዚሽን በፈለገችው ፖዚሽን ስትሆን እኛ የተሻለ ሰርቢስ ሰተናል ብለን እናስባለን ደግሞ የተሻለ ሰርቢስ አልሰጠንም ብለን የምናስበው አሁን አንዲት እናት በመጋረጃ መውለድ እፈልጋለሁ ብትል የመጋረጃ እጥረት ስላለ ያለመጋረጃ ነው የምናዋልዳት እና እሱ እሱ ነገር ስታይ ሲሳቀቁ ካለዉ ከካልቸሩም በተለይ ሞስሊም እናቶች በዚህ ፍራስተሬት ያደርጋሉ እና እሱን እሱን እንትን አንል ስንል ሰርቢስ አልሰጠንም ብለን በኛ እናሰባለን ከባለሙያው አንጻር ደግሞ ይኖራል የናቶች ሰርቢስ በአንዴ ገብቶ ባንዴ ሚባል ነገር አደለም ሰዓት ሚጨርስ ነው ጊዜ እንትን ይላል እንደነገርኩህ ድካም በራሱ ሙሉ ሰርቢስ እንሳንሰጥ ያደርገናል ለምሳሌ ሌሊት ሶስት ሚድዋይፍ ነው የሚያደር ሶስት ሚድዋይፍ አስር እናት አስራሁለት እናት ቢወልድ ያከድካም ሚመጣ እንትን አለማለት አለ አለመረዳት ነገር ይኖራል ማታ መሆኑ ያው አንድ እንትነው ያው ሎድ አለ ጀነራል ሆስፒታል እንደመሆኑ የእናቶች ፍሰት ከሄልዝ ቸንተር ወደኛ ሞር የበዛ ስለሆነ ከድካም አንጻር እንትን ላንል እንችላለን አንድ ሰው ጥሩ ሲሰራ የተሻለነገር ማበረታቻ ያስፈልገዋል ካልሰራ ደግሞ ለሚን ተብሎ መጠየቅ አለበት እንደኛ ሆስፒታል ይሄ ነገር የተለመደ አደለም ሚሰራዉም አይበረታታም ማይሰራውም ለምን አይባልም እሱእሑ ነገር በርግጥ እንትን ይላል እንዳንድ ምክኒያት ሊሆን ይችላል ሰምበዲ የሆነ ነገር አድርጎ ካልተጠየቀ ለምን ሚባል ነገር

ጠያቂ:በአንተ አስተሳሰብ አንዲት እናት በምትወልድበት ሰዓት በአክብሮት አገልግሎቱን እንድታገኝ/እንዳታገኝ ሊያደርጉ የሚችሉ ተያያዥ ጉዳዮች ምን ምን ሊሆኑ ይችላሉ ብለህ ታምናለህ? እንዴት?

ተሳታፊ: ጥሩ ሰርቢስ ላለመስጠት ሌላው ምንድን ነው ለምሳሌ አንዲት እናት ልጇ ቢታፈንባት እኔ በዚህኛው መተኛት እፈልጋለሁ መቀመጥ እፈልጋለሁ መቆም እፈልጋለሁ ብትል ያንን ነገር እንትን አንለም ማለት ሴፍ ሳይድ የሆነዉን ለእናትዮዋም ለልጇም የምንከተል እነሱ ደግሞ በመቆሟ ወይምሚፈልገዉን ፖዚሽን እኛለህጻኑ ባለመያዛቸዉ የሚመጣ ችግር ያለ ስለማይመስላቸዉ እሱ እራሱ እኝ ጥሩ ነገር እንዳላደረኝላቸዉ ይገምታሉ እሱ እሱ ንገር አንድ ተግዳሮት ነው ሰው ያለዉ ህመሙን ነው ፊል ሚደርገው እና ከዛ ከዛ አንጻር እሱም አንድ ችግር ነው ቢለን እናሰባለን

ጠያቂ:የወሊድ አገልግሎቱን የሚሰጠው/ባለሙያው አንዲት እናት በምትወልድበት ሰዓት ስነ-ምግባር በጎደለው ሰብዕና ሲያስተናግድ ተይዞ ተጠያቂ የሚሆነው እንዴት ነው?

ተሳታፊ: ለምሳሌ የሆነ ነገር ሃፕን ሲሆን ተከታትሎ የራሱ የሆነ ኮሚቴ የለዉም ማለት አንድ ነገር ሃፕን ሲሆን ያው የቅርብ ሃላፊህ ለምን እዲህ ሆነ በቃበቃ ከዚህ የዘለለ ነገር ስለሌለ እንዳንድ

ጠያቂ:ሳናነሳው የቀረ ነገር ግን ለጥናቱ ይጠቅማል የምትለው/ይዉ ሀሳብ ካለ የመጨረሻ እድል ልስጥህ/ሽ?

ተሳታፊ: አንደኛ ነገር ሲአር ሲ ትሬኒግ ቢኖር ባለሙያው በየጊዜዉ ለነገሮች አብዴት ቢሆን ጥሩ ነው ሁለተኛ ሁለተኛ ነገር በየ ተቋማቱ ክትትል ቢደረግ ምን ተሰራ ምን ተደረገ በቅርብ ክትትል ቢኖር በሶስተኛ ደርጃ ደግሞ ግባቶች ቢሟሉ እነዚህ የጠቀስናቸው ቢሟሉ የእናቶች አገልግሎት የተሻለ ሰቢስ ሊኖር ይችላል ብየ አስባለሁ
