# Supplementary material for: Respectful maternity care and associated factors among mothers who gave birth at public health institutions in Debre Tabor town, Northwest Ethiopia: a mixed-methods study
Source: Front Glob Womens Health. 2025 Jan 23;6:1513906. doi: 10.3389/fgwh.2025.1513906 (PMC11798984; doi:10.3389/fgwh.2025.1513906)
Supplement: Supplementary file 3 [file Datasheet3.pdf]

Type of data: Qual

Date of interview: 24/04/2016

Participant order: KII1

Duration of audio time: 07:06 second

Interviewer: Do you share your thoughts on what respectful maternity care means to you? In your opinion, how would you rate the quality of respectful maternity care service provided in this health facility? Can you explain the idea behind your answer?

Participant: "Reasons for incomplete completion can be attributed to various factors. Firstly, in terms of infrastructure, there is a lack of privacy screens to protect patients' confidentiality. This deficiency in privacy protection is a significant concern. When a mother comes to give birth, the entire family of the villagers gathers around, and it is the responsibility of the healthcare provider to manage the situation. However, due to the absence of privacy screens, the examination process becomes challenging as the family members tend to interfere and gather around, hindering the healthcare provider's ability to conduct a thorough physical examination. Moreover, there exist imbalances between the number of healthcare professionals and the patient population. This disparity further complicates the situation. Additionally, another contributing factor is the parents' reluctance to heed the advice of experts. This lack of adherence to professional guidance can lead to suboptimal outcomes for both the mother and the baby. Furthermore, the nighttime poses additional challenges in our hospital. During this period, the number of healthcare professionals decreases, while the number of mothers in need of care increases. This imbalance creates fatigue among healthcare providers, which can result in decreased efficiency and potential boredom. Consequently, the quality of care provided to mothers may be compromised. Another issue that hinders the completion of tasks is the lack of recognition and promotion for healthcare professionals. Despite serving for eight years, I received no promotions or educational opportunities. This dissatisfaction with the lack of career advancement is further exacerbated by the relatively low salary of approximately 8000 Birr. Comparing myself to individuals of the same age in other professions, this dissatisfaction becomes more apparent. As a result, the diminishing love for the profession gradually affects the quality of service provided to mothers. Dissatisfaction with life and work can lead to reduced dedication and commitment. It is crucial to address these underlying issues to ensure the provision of complete and comprehensive care to mothers in need."

Interviewer: Have you ever found yourself in a situation where you felt like you acted in a way that respected women during childbirth? If so, can you elaborate on when and how it happened?

Participant: "In cases of fetal distress and maternal bleeding, it may be difficult to manage the situation. The healthcare provider must prioritize the mother's safety and prevent excessive bleeding, while also ensuring that the baby is not harmed".

Interviewer: From your perspective, do you think the respectful maternal care issue has been adequately addressed? Can you elaborate on why and how you came to this conclusion?

Participant: "In our hospital, administrative rules often lead to dissatisfaction with the profession due to the lack of positive responses to our inquiries"

"I have not seen any accountability in the CRC yet. Even if they cause a violation of rights or abuse, they will not be held liable unless it results in harm to human rights. I haven't seen any action taken in cases where there is no obvious harm like stillbirth or UFD. In the CRC, I have not witnessed individuals being asked whether a case has resulted in insult or violation of their rights."

Interviewer: Lastly, do you have any additional ideas or thoughts on the topic that you believe is crucial and should be discussed?

Participant "If we study the gap and establish accountability, fatigue can be reduced by issuing a number standard. Additionally, addressing any other issues raised by the immediate supervisor, providing education opportunities, and offering benefits can help fix these things."

Type of data: Qual

Date of interview: 24/04/2016

Participant order: KII2

Duration of audio time: 11:31 second

Interviewer: Can you share your thoughts on what respectful maternity care means to you? In your opinion, how would you rate the quality of respectful maternity care service provided in this health facility? Can you explain your reasoning behind your answer and how you arrived at it?

Participant: “All mothers who come to us receive respectful health care. That is, it is assumed that they will get the service they want when they want it in our hospital”.

Interviewer: What do you believe are the key factors that impact the provision of respectful delivery service? Can you explain in detail?

Participant: “A respectful maternity care (CRC) approach may not be applicable in situations where there is an overload of cases and an imbalance in the number of professionals available. For instance, a professional may be required to attend to two or three mothers at once. Such situations may lead to fatigue, especially if it occurs at night when the professional is supposed to be sleeping”.

“Sometimes when a mother comes to give birth, about ten attendants may come with her; to keep her privacy, you might instruct a companion to leave the room, but might not do so. There is a shortage of security personnel. This situation makes mothers without proper care; we do everything for the sake of the mother and fetus”

Interviewer: Have you ever found yourself in a situation where you felt like you acted in a way that respected women during childbirth? If so, can you elaborate on when and how it happened?

Participant: “Providing respectful maternity care can be challenging under certain circumstances. For example, if a mother experiences fetal distress, she may need to lie on her back to monitor the fetus. In such a situation, we may not be able to respect her preferred position to save the lives of the fetus fetus”

Interviewer: From your perspective, do you think that the issue of respectful maternal care has been adequately addressed? Can you elaborate on why and how you came to this conclusion?

Participant: "You don't work for someone else, you work for yourself. Your job is your boss. If something happens with your immediate boss, you may feel trapped and not be able to properly take care of your mother.".

“I don't believe accountability is beneficial because the professional may not apply respectful maternity care to the recipient of the service.”

Interviewer: Lastly, do you have any additional ideas or thoughts on the topic that you believe is crucial and should be discussed?

Participant: “This type of issue can arise from expert fatigue, an overwhelming caseload, or a lack of proportional representation of professionals and mothers. Every mother needs to receive the appropriate services.”

Type of data: Qual

Date of interview: 24/04/2016

Participant order: KII3

Duration of Audio time: 08:35 second

Interviewer: Can you share your thoughts on what respectful maternity care means to you? In your opinion, how would you rate the quality of respectful maternity care service provided in this health facility? Can you explain your reasoning behind your answer and how you arrived at it?

Participant: "When a professional takes three or four beds, we may not be able to attend to all the mothers when they need us. If we attend to them when they don't need us, they may not be willing to receive our services and see us as intrusive. At night, fatigue may make it difficult for us to provide comprehensive CRC services. The professional in this hospital and the case at hand are unrelated, and there could be disagreements with your boss. When you do not receive the benefits you were promised or when there are family problems, lack of material supply it can add to the stress."

Interviewer: Have you ever found yourself in a situation where you felt like you acted in a way that respected women during childbirth? If so, can you elaborate on when and how it happened?

Participant: “We may not be able to prioritize the mother's needs in cases of fetal distress or complications”

Interviewer: From your perspective, do you think that the issue of respectful maternal care has been adequately addressed? Can you elaborate on why and how you came to this conclusion?

Participant: "I don't view discipline in the same way as others do. My focus is on fulfilling my responsibilities. If there are issues with her fetus or mother, it falls upon us to take care of them. Rather than thinking about what's right or wrong, our priority is to prevent any complications from arising."

Interviewer: Lastly, do you have any additional ideas or thoughts on the topic that you believe is crucial and should be discussed?

Participant: “As you know, being a midwife is a serious job with a heavy workload, responsibilities, and risks. However, there are limited educational opportunities and paths for advancement”.

Type of data: Qual

Date of interview: 24/04/2016

Participant order: KII4

Duration of Audio time: 12:10 second

Interviewer: Can you share your thoughts on what respectful maternity care means to you? In your opinion, how would you rate the quality of respectful maternity care service provided in this health facility? Can you explain your reasoning behind your answer and how you arrived at it?

Participant: "The level of respectful maternal health service provision in our institution is average. The provision of respectful maternal health services varies from one person to another, but I cannot say that we have provided enough of these services. There is inconsistency in the provision of respectful maternal health services in our institution, and we have yet to reach the level of provision we aim to achieve."

Interviewer: What do you believe are the key factors that impact the provision of respectful delivery service? Can you explain in detail?

Participant: “In our area, there are many mothers who are uneducated and have difficulty communicating with us. Sometimes they want to give birth as soon as they arrive at the hospital and may not understand the procedures. They may also have specific requests or demands, which can lead to misunderstandings. Additionally, we have a heavy workload, which may make it challenging to provide the necessary level of attention and care to all mothers.”

“Mothers often come to the hospital for childbirth after experiencing false labor at home. They may feel like they are ready to give birth as soon as they arrive, but the time spent at home can sometimes lead to misunderstandings, boredom, and changes in behavior”.

"When an employee experiences disagreements with colleagues and the institution does not provide opportunities for promotion or education, they may begin to dislike their job and become bored. On the other hand, if a person is required to handle multiple cases simultaneously, it could lead to a decrease in the quality of service provided. In such a situation, the service provided may not be respectful."

“One issue is that there is an insufficient supply of goods, so people buy them from abroad. Parents who come to visit find it difficult to purchase items because they expect the service to be free. This financial obstacle prevents you from pursuing the work you desire, which is a major barrier to providing respectful maternity care”

“The lack of supervision by superiors results in staff being out of control which hinders the provision of respectful maternity care”

Interviewer: Have you ever found yourself in a situation where you felt like you acted in a way that respected women during childbirth? If so, can you elaborate on when and how it happened?

Participant: “When fetal distress arises, I must monitor the fetus's heartbeat regularly. In situations where the mother is experiencing pain and it becomes difficult to count the fetal heart rate, I will prioritize positioning her in a way that protects the fetus rather than respecting her rights”

“Mothers who are in labor for the first time sometimes feel like they have not been treated well. After providing them with a bed and conducting an initial examination, we typically check on them every hour unless they have an obstetric issue. However, we tend to check on mothers who are co-sleeping every thirty minutes or every fifteen minutes, which can make first-time mothers feel neglected. As a result, mothers in the first stage of labor may feel like they have been abandoned”

Interviewer: From your perspective, do you think that the issue of respectful maternal care has been adequately addressed? Can you elaborate on why and how you came to this conclusion?

Participant: “If my immediate superior does not respect and value me, I will lose respect for those beneath me and for the people I serve”.

“In situations where there is no accountability, people often neglect their behavior. However, being held accountable motivates them to pay more attention”.

Interviewer: Lastly, do you have any additional ideas or thoughts on the topic that you believe is crucial and should be discussed?

Participant: "When a mother brings someone else to the childbirth, it can sometimes hinder us from providing proper services. As health professionals, we may become distracted or interrupted by the presence of a third party, which could lead to inappropriate actions or decisions being made. Therefore, it is important to maintain a professional and focused environment that ensures the safety and dignity of both the mother and her child."

Type of data: Qual

Date of interview: 25/04/2016

Participant order: KII5

Duration of audio time: 06:14 second

Interviewer: Can you share your thoughts on what respectful maternity care means to you? In your opinion, how would you rate the quality of respectful maternity care service provided in this health facility? Can you explain your reasoning behind your answer and how you arrived at it?

Participant "The reason why some cases may not receive appropriate service is that the service providers may be overwhelmed with a high number of cases. Additionally, some service providers may not receive adequate training, which could result in subpar service. Another issue could be the absence of a screen while listening to the fetal heartbeat, which could result in the provision of inappropriate service."

Interviewer: Have you ever found yourself in a situation where you felt like you acted in a way that respected women during childbirth? If so, can you elaborate on when and how it happened?

Participant: "In some cases, a patient's life or well-being may be in danger, and you may need to take action even if it goes against the patient's wishes. For example, if a patient is bleeding heavily, you may need to intervene to save their life, even if the patient does not want you to do so. It is important to strike a balance between respecting the patient's autonomy and taking the necessary steps to protect their health"

Interviewer: From your perspective, do you think that the issue of respectful maternal care has been adequately addressed? Can you elaborate on why and how you came to this conclusion?

Participant: "Accountability is crucial for effective functioning, but its absence cannot necessarily be equated with failure. Regardless, professional standards must remain the focus of any hospital or institute, establishing a ratio of accountability for both actions taken and not taken would ensure better service for patients."

Interviewer: Lastly, do you have any additional ideas or thoughts on the topic that you believe is crucial and should be discussed?

Participant: If patients are informed of their rights, they should also know their obligations.

Type of data: Qual

Date of interview: 25/04/2016

Participant order: KII6

Duration of audio time: 05:50 second

Interviewer: Can you share your thoughts on what respectful maternity care means to you? In your opinion, how would you rate the quality of respectful maternity care service provided in this health facility? Can you explain your reasoning behind your answer and how you arrived at it?

Participant: “Respectful work is not always perfect, but striving for it yields positive results” “There can be several reasons, however, For instance, an increase in workload may be a reason”.

Interviewer: Have you ever found yourself in a situation where you felt like you acted in a way that respected women during childbirth? If so, can you elaborate on when and how it happened?

Participant: “During childbirth, if a woman experiences bleeding and fetal distress, the provider may prioritize interventions to save the fetus and prevent bleeding. This may take precedence over the principles of respectful maternity care”.

Interviewer: From your perspective, do you think that the issue of respectful maternal care has been adequately addressed? Can you elaborate on why and how you came to this conclusion?

Participant: "I believe that the lack of accountability is one of the contributing factors to the problem. People should be held accountable for their actions, especially when they are disrespectful or hurtful towards someone else. If they are not held accountable, it can lead to further harm and negative consequences. I think it's important for people to take responsibility for their actions, learn from them, and work towards repairing any damage that has been done, especially in their relationships with others."

Interviewer: Lastly, do you have any additional ideas or thoughts on the topic that you believe is crucial and should be discussed?

Participant: “Health professionals should be provided with proper training and modules for RMC”

Type of data: Qual

Date of interview: 25/04/2016

Participant order: KII7

Duration of audio time: 11:08 second

Interviewer: Can you share your thoughts on what respectful maternity care means to you? In your opinion, how would you rate the quality of respectful maternity care service provided in this health facility? Can you explain your reasoning behind your answer and how you arrived at it?

Participant: “Health facilities were not always fully equipped with all necessary equipment like; screens, there is a lack of protective equipment often when you are splashed with blood, which might result in disrespectful care”

Interviewer: Lastly, do you have any additional ideas or thoughts on the topic that you believe is crucial and should be discussed?

Participant: “Various problems prevent us from giving respectful maternity care, administrative problems like supply and there is also lack of training on respectful maternity”

Type of data: Qual

Date of interview: 25/04/2016

Participant order: KII8

Duration of audio time: 09:23 second

Interviewer: Can you share your thoughts on what respectful maternity care means to you? In your opinion, how would you rate the quality of respectful maternity care service provided in this health facility? Can you explain your reasoning behind your answer and how you arrived at it?

Participant: “I believe respectful maternity care services are good, but there may be room for improvement in certain situations”.

Interviewer: What do you believe are the key factors that impact the provision of respectful delivery service? Can you explain in detail?

Participant: “One of the toughest issues is the problem of supply, which poses a challenge to the healthcare sector. In addition, there is a shortage of professionals, which further exacerbates the problem. For instance, in the maternity ward of a hospital, there are only seven beds available for expectant mothers from the time they arrive until they give birth, but there are only three professionals available to attend to them. The remaining personnel comprises students and university staff, who might not have the required expertise. Hence, the shortage of professionals poses a significant challenge in providing respectful maternal health services”.

“Because there is a lack of a screen, we did the physical examination without a screen, in this situation especially Muslim mothers get frustrated “

"I feel fatigued, especially at night, because there are only three midwives available. On average, there are ten to twelve mothers who need to give birth, and this overwhelms me, making it difficult to provide respectful care. Furthermore, there is no support or encouragement provided to the staff"

Interviewer: Have you ever found yourself in a situation where you felt like you acted in a way that respected women during childbirth? If so, can you elaborate on when and how it happened?

Participant: “Childbirth can be challenging, and women experience a lot of pain. While we prioritize the health of the mother and fetus, we may not always respect her choice of position.”

Interviewer: From your perspective, do you think that the issue of respectful maternal care has been adequately addressed? Can you elaborate on why and how you came to this conclusion?

Participant: “If healthcare professionals receive appropriate training and institutions implement monitoring systems, respectful maternity services can be ensured”.
