# Supplementary figures and images for: Respectful maternity care and associated factors among mothers who gave birth at public health institutions in Debre Tabor town, Northwest Ethiopia: a mixed-methods study
Source: Front Glob Womens Health. 2025 Jan 23;6:1513906. doi: 10.3389/fgwh.2025.1513906 (PMC11798984; doi:10.3389/fgwh.2025.1513906)

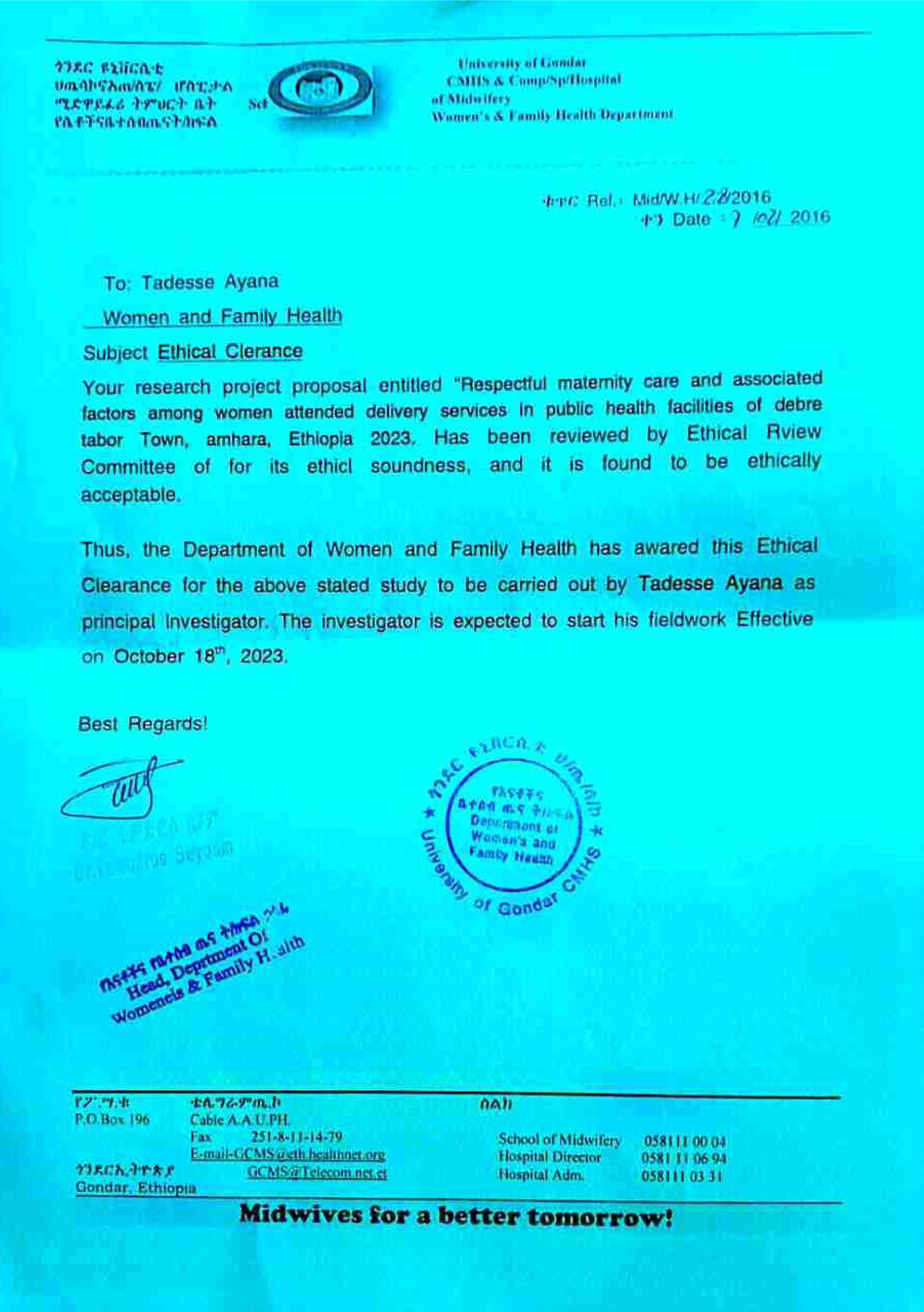

Supplement: Supplementary file 12 [file Image1.tif]
